# A biomolecular perspective on mobile pastoralism and its role in wider socioeconomic connections in the Chalcolithic South Caucasus

**DOI:** 10.1016/j.isci.2025.112544

**Published:** 2025-05-02

**Authors:** Mariya Antonosyan, Gwendoline Maurer, Satenik Mkrtchyan, Kseniia Boxleitner, Mariam Saribekyan, Anahit Hovhannisyan, Laura Furquim, Freg Stokes, Ruben Davtyan, Arsen Bobokhyan, Karen Azatyan, Jana Ilgner, Sabine Reinhold, Ellery Frahm, Robert Spengler, Patrick Roberts, Noel Amano, Levon Yepiskoposyan

**Affiliations:** 1 Department of Archaeology, Max Planck Institute of Geoanthropology, 07743 Jena, Germany; 2 Department of Coevolution of Land Use and Urbanisation, Max Planck Institute of Geoanthropology, 07743 Jena, Germany; 3 School of History, Archaeology and Religion, Cardiff University, CF10 3AT Cardiff, UK; 4 Institute of Molecular Biology, National Academy of Sciences, Yerevan 0014, Armenia; 5 Domestication and Anthropogenic Evolution Research Group, Max Planck Institute of Geoanthropology, 07743 Jena, Germany; 6 Institute of Archaeology and Ethnography, National Academy of Sciences, Yerevan 0025, Armenia; 7 Smurfit Institute of Genetics, Trinity College Dublin, Dublin D02 VF25, Ireland; 8 Department Structural Changes of the Technosphere, Max Planck Institute of Geoanthropology, 07743 Jena, Germany; 9 State Office for Heritage Management and Archaeology Saxony-Anhalt – State Museum of Prehistory Halle (Saale), 06114 Halle (Saale), Germany; 10 Yeghegnadzor Regional Museum, Yeghegnadzor 3601, Armenia; 11 German Archaeological Institute, 14195 Berlin, Germany; 12 Council on Archaeological Studies, Department of Anthropology, Yale University, New Haven, CT 06511, USA; 13 Anthropology Division, Peabody Museum of Natural History, Yale University, New Haven, CT 06511, USA

**Keywords:** Paleobiology, Anthropology, Archeology

## Abstract

Mobile pastoralism is widely evoked when discussing technological developments, resource procurement, *trans*-regional interactions, and exchange networks in the South Caucasus. In this study, we conduct a comprehensive multiproxy investigation of faunal and botanical remains from the Middle to Late Chalcolithic in southern Armenia, at the high altitude Yeghegis-1 site, to directly assess herd mobility and human subsistence practices. Our findings indicate that, alongside intensified interregional connectivity, the inhabitants practiced a rather sedentary form of multi-resource pastoralism, while maintaining herds at the site year-round. These results complement and expand upon models of pastoral mobility and its perceived crucial role in sustaining inter- and intra-regional connectivity. We argue that alternative models of increased intra-regional connectivity, focused on exchange between different specialized settled economies, need to be considered and further research is essential to unravel the complex interplay between subsistence, trade, and socio-economic dynamics.

## Highlights

• Integration of Zooarchaeology, archaeobotany, ZooMS, and stable isotope data
• Subsistence economy focused on sheep and goats herding
• Low-investment plant cultivation and opportunistic foraging
• Settled pastoral system with year-round reliance on local highland resources

## Graphical abstract

**Figure:**
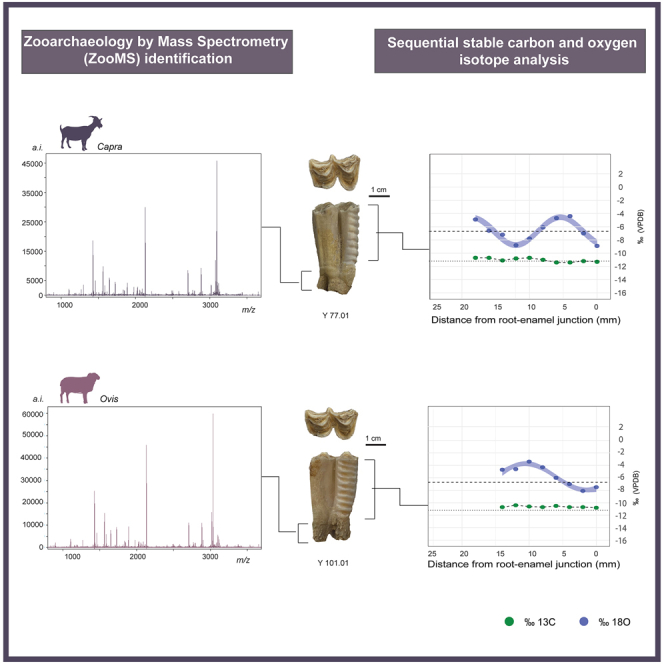

## Introduction

Mobile pastoralism in Southwest Asia including the Caucasus has variously been seen as an adaptation to climate stresses, marginal environments, resource availability, or socio-political transformations,^1^^,^^2^^,^^3^^,^^4^^,^^5^^,^^6^^,^^7^^,^^8^ while at the same time it has been traditionally considered as operating at the “fringes” or outside of major social and environmental changes, such as those linked to the emergence of agriculture and urbanisation.^9^^,^^10^ Drawing heavily upon ethnographic parallels,^9^^,^^11^ long-distance pastoralists are often framed as carriers of technologies and “cultures”, connecting core areas to so-called margins and peripheries.^1^^,^^2^^,^^9^^,^^12^^,^^13^ In many regions, including the Caucasus, this topic has been the focus of studies investigating social developments.^5^^,^^14^^,^^15^^,^^16^ This has resulted in reconsideration and readjustment of the interactions between sedentary and mobile sectors of the population. Cutting edge bioarchaeological methods open up completely new possibilities for discussing the mobility radii of humans and animals, investigating the fundamentals of group diets and looking at long-term processes.^16^^,^^17^ Much attention has been focused on the Fertile Crescent and Mesopotamia, since socioeconomic changes in these regions (e.g., food production and the emergence of urban city-states) have been considered to have had major, lasting consequences that diffused (or demographically expanded) across the wider region.^2^^,^^3^ Yet, not only have these processes been proven to be far more complex, nonlinear, and diverse in these regions, but the fringes of these supposed core regions are also coming into the focus of research, complementing and diversifying many of the previous assumptions.^2^^,^^5^^,^^9^^,^^15^^,^^16^^,^^18^^,^^19^^,^^20^ The South Caucasus region, situated at the cultural and economic crossroads between the Near East and wider Eurasia,^21^^,^^22^ provides a perfect case study, as the region has often been considered peripheral in historical and archaeological discourse,^23^ and pastoral mobility has frequently been used to explain cultural transformations. However, narratives of pastoral mobility in the Caucasus are often oversimplified and generalized, often being reduced to a single system for each chronological period.^24^^,^^25^^,^^26^

**Figure 1:**
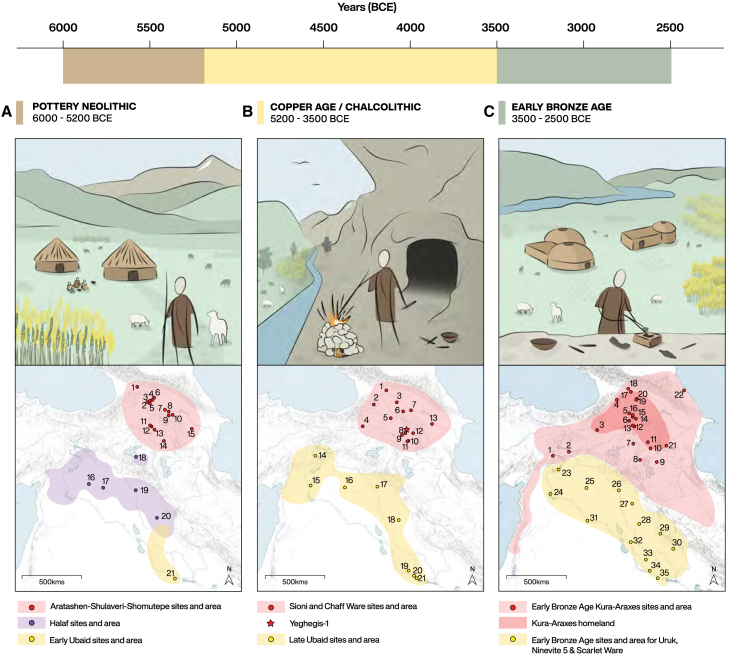
Visual summary of current knowledge and prevailing narratives of socioeconomic change in the Neolithic, Chalcolithic, and Early Bronze Ages in the Southern Caucasus (A) The distribution of Neolithic Aratashen-Shulaveri-Shomutepe sites (1 Darkveti; 2 Shulaveris Gora; 3 Gadachrili Gora; 4 Imiris Gora; 5 Arukhlo; 6 Khramis Did Gora; 7 Shomutepe; 8 Hacı Elamxanlı Tepe; 9 Göytepe; 10 Mentesh Tepe; 11 Aratashen; 12 Aknashen; 13 Masis Blur; 14 Kültepe; 15 Kamiltepe). (B) Distribution of Chalcolithic sites (1 Berikldeebi; 2 Bavra-Ablari; 3 Dzedzvebi; 4 Sioni; 5 Sos Hoyük; 6 Tsaghkunk; 7 Getahovit; 8 Mentesh Tepe; 9 Areni-1; 10 Ovçular Tepe; 11 Godedzor; 12 Leilatepe; 13 Nakhchivan Tepe; 14 Kultepe; 15. Alikemek Tepesi). (C) Distribution of Early Bronze Age Kura-Araxes sites (1 Gudabertka; 2 Natsargora; 3 Chobareti; 4 Kiketi; 5 Koda; 6 Samshvilde; 7 Karnut; 8 Gegharot; 9 Tsaghkahovit; 10 Harich; 11 Aparan III; 12 Talin; 13 Sos Hoyük; 14 Mokhrablur; 15 Shengavit; 16 Köhne Shahar; 17 Kültepe (Nakhichevan); 18 Kul Tepe (Hadishahr); 19 Agarak; 20 Kohne Pasgah Tepesi; 21 Haftavan Tepe; 22 Yanik Tepe).

The Pottery Neolithic in the South Caucasus has been defined by permanent villages with fully sedentary economies, relying on domesticated plants and animals.^27^^,^^28^^,^^29^^,^^30^^,^^31^^,^^32^^,^^33^^,^^34^ However, a more varied picture is now emerging.^35^ The early development of pastoral mobility practices during the Neolithic is regarded as a significant factor in the intensification of management strategies and the resulting social transformations.^36^^,^^37^^,^^38^ Scholars have argued that during the Chalcolithic the Caucasus witnessed a shift toward a more mobile lifestyle, transforming into a dynamic hub of innovation and connectivity.^25^^,^^38^^,^^39^^,^^40^^,^^41^^,^^42^^,^^43^^,^^44^ This transformation is suggested to be linked to the exploitation of highland resources and significant technological developments, such as the emergence of early mining and metallurgy.^21^^,^^25^^,^^41^^,^^42^^,^^45^^,^^46^^,^^47^^,^^48^^,^^49^^,^^50^ Existing evidence used to discuss the expansion of seasonal pastoral use of highland areas focuses on the fact that many of the Chalcolithic sites are located at high altitudes, for which year-round use has been excluded due to climatic conditions (see Figure 1 for an overview of regional narratives and the distribution of key archaeological sites.).^24^^,^^50^^,^^51^ Even if this is true for some places,^52^ the question arises as to whether mobile forms of economy were the only possible forms in the highlands.

High degrees of pastoral mobility also remain a prominent variable in models explaining the connectivity and spread of the Early Bronze Age Kura–Araxes culture throughout Southwest Asia.^17^^,^^53^^,^^54^^,^^55^ Mobile pastoralism is proposed as a key factor underpinning the expansion of inter- and intra-regional obsidian networks and cultural exchange with Mesopotamia, Iran, Anatolia, and the North Caucasus during the Chalcolithic.^17^^,^^22^^,^^24^^,^^39^^,^^40^^,^^51^^,^^56^^,^^57^^,^^58^^,^^59^^,^^60^^,^^61^^,^^62^^,^^63^ Archaeologists working in the Middle and Late Bronze Age Caucasus have argued that pastoral mobility is also an instrumental factor in the emergence of political complexity in the region.^64^^,^^65^^,^^66^

Nevertheless, the number of studies explicitly and directly addressing the issue of human and animal mobility is still limited. Most recent studies often are based on settlement data and use more traditional arguments from archaeology, archaeozoology, and archaeobotany.^23^^,^^31^^,^^34^^,^^54^^,^^66^^,^^67^ Therefore, our understanding of pastoralism and mobility practices in the Chalcolithic of the Caucasus and their relationship to broader regional social and economic changes is somewhat biased. Smaller or less intensive sites at high altitudes, characterized by more ephemeral cultural layers have been interpreted as short-lived occupations by highly mobile users.^24^^,^^29^^,^^37^^,^^38^^,^^44^^,^^51^ Recent advances in biomolecular techniques and cross-disciplinary approaches enable us to unlock previously inaccessible information on pastoral land use and mobility, permitting reassessments of long-held views using direct proxies. Zooarchaeology and archaeobotany, when combined with biomolecular approaches such as collagen fingerprinting, provide more refined insights into human resource use.^68^ Meanwhile, stable isotope analyses applied to herd animals can yield deeper understanding of past herding strategies.^69^ In contrast to the North Caucasus, where long-term and extensive studies on the analysis of stable isotopes in humans and animals are already available,^70^^,^^71^^,^^72^^,^^73^ the studies in the South Caucasus have so far been regionally and temporally limited. Studies exist on Neolithic sites,^35^^,^^74^^,^^75^ some Chalcolithic sites,^39^^,^^41^^,^^67^ and on sites of the Early and Late Bronze Age.^65^^,^^76^^,^^77^^,^^78^

To complement existing knowledge and expand our understanding of lifeways during this key period of human history and social and economic interactions in the Caucasus, we initiated the archaeological exploration of high altitude Yeghegis-1 rock shelter. Our previous research efforts at the site focused on obsidian sourcing and uncovered that communities exploited a range of raw material sources, many of which are located in mountainous zones. At the same time, the analysis revealed an increase in obsidian diversity through time suggesting shifts in land use and, in turn, social connections across the wider region.^79^ However, the mechanisms by which these connections were sustained and the extent to which pastoral mobility contributed to their maintenance remain to be clarified.

This paper investigates the lifeways of Chalcolithic pastoralists inhabiting Yeghegis-1, with a focus on herd composition, livestock management strategies, and role of cultivation in the economy at the site, while incorporating diverse lines of evidence. Here, we apply radiocarbon dating and chronological Bayesian modeling of obtained dates to refine the chronology of the site. We assess the taxonomic composition of fauna, using a combination of traditional zooarchaeological and molecular collagen fingerprinting approaches. We analyze stable carbon and nitrogen isotopes of bone collagen and stable carbon and oxygen isotopes of bulk tooth enamel from faunal remains to explore life histories of animals consumed at Yeghegis-1. Additionally, we employ stable carbon and oxygen isotope analysis of sequentially sampled tooth enamel from Caprines to reveal how communities maintained herds in the topographically varied region. The sequential sampling along high-crowned teeth of herded domesticates can offer insights into vertical transhumant pasture strategies used by the communities.^64^^,^^73^^,^^80^ We report archaeobotanical data to provide information on cultivated and wild plants utilized by the inhabitants of the site.

Our research has yielded insights into pastoral management practices, human diet, and economic connections, challenging the prevailing narrative that Chalcolithic groups were predominantly mobile pastoralists. We suggest that alternative variables for increased intra-regional connectivity need to be considered, such as demographic growth and expansion, trade and exchange systems, and technological diffusion, rather than relying on the single lever of pastoral mobility. Further research, including additional sites and a wider range of evidence, is essential to unravel the complex interplay between human subsistence, trade, and socioeconomic dynamics.

## Results

### Site and chronology

**Figure 2:**
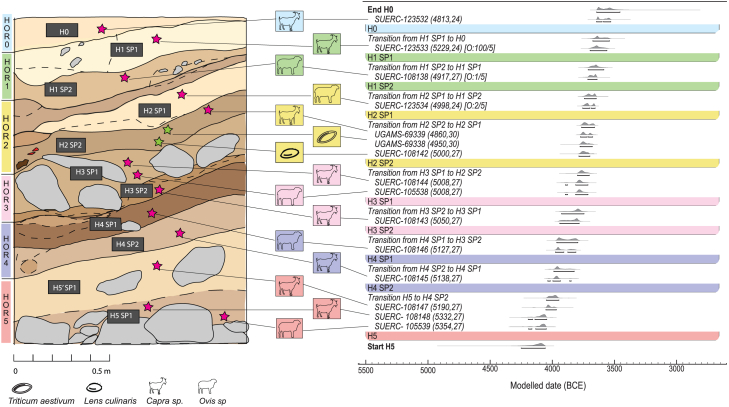
The stratigraphy and chronological Bayesian modeling of the site constructed from calibrated ^14^C dates on animal bones (identified via ZooMS) and seeds (all dates are detailed in Table S1) The modeling used the IntCal20.14c calibration curve. Source: OxCal v4.4.4. r:5. The site sequence encompasses Horizons (H0 to H5) with transition boundaries between spits and horizons. Confidence intervals are expressed in 95.4% range bars.

Yeghegis-1 (N39°51′52.72″, E45°20′41.52″) is a basalt rock shelter situated on the northern bank of the Yeghegis River, in Vayots Dzor Province, Armenia, at an elevation of 1,500 m above sea level. The first brief scientific description of the site was completed in 2020 by our team, followed by a preliminary archaeological survey in 2021 and large-scale excavations in 2022 and 2023. The material presented here was recovered from Trench 2 excavated during the 2022 field season. The excavations revealed a sequence of distinct, undisturbed occupational layers (Horizons 1–5), yielding abundant animal remains, lithic artifacts, ceramic sherds, and copper and bone artifacts. Previous dating efforts at the site revealed a 600-year occupation ranging from the end of the Middle Chalcolithic (ca. 4100–4000 cal BCE) through the end of the Late Chalcolithic (ca. 3600–3500 cal BCE) in this region^79^^,^^81^ (Figure 2). Newly obtained ^14^C dates by directly dating archaeobotanical seeds fit well into the established chronology of the site. Lentil (*Lens culinaris*) and free-threshing wheat (*Triticum aestivum*) remains recovered from Horizon 2 returned ^14^C dates of 3659–3626 cal BCE and 3783–3649 cal BCE, respectively.

### Macrobotanical remains

Archaeobotanical analysis recorded the presence of 1,151 charred seeds or large seed fragments in total, spanning all Horizons and 7,919 carbonized wood fragments (>2 mm; Table S2). Agricultural crops at the site are represented by barley (*Hordeum vulgare*; *N* = 5) and wheat (*Triticum aestivum*; *N* = 4), as well as 96 fragments of cereal grains, for which further taxonomic identification was restricted due to the high fragmentation rate. Pulses in the site are represented by lentil (*Lens culinaris*) cotyledons (*N* = 10) and one likely chickpea (*Cicer arietinum*) fragment (Figure S1). Oil seed crops were absent, with the exception of a single seed of flax (*Linum usitatissimum*), which may be either wild or cultivated. Plant parts belonging to fruit (*N* = 107) and nut trees (*N* = 3) add up to less than 12% of the total identified plant remains, with hackberry (*Celtis australis*; *N* = 94), Russian olive (*Elaeagnus angustifolia*; *N* = 1), *Prunus* subg. *Padus* (*N* = 8), and grape (*Vitis vinifera*; *N* = 1), all of which grow wild around the site and are native to the region.^82^ Seeds of wild/weedy plants comprise the majority of identified charred plant remains. Amaranthaceae (*N* = 280), *Galium* sp. (*N* = 237), and *Onopordum acanthium* (*N* = 121) dominate the plant assemblage, constituting over 55% of the seed count from the site. Weedy zoochoric species associated with anthropogenic disturbance, include *Medicago/Melilotus* spp. (*N* = 7) and wild Fabaceae seeds (*N* = 19), as well as *Galium* sp., *Lithospermum arvense*, and Polygonaceae. Other plants such as *Neslia paniculate*, species of Asteraceae, Polygonaceae, and Amaranthaceae, belong to a ruderal group of taxa, which is largely redundant from the previous group, as weedy and ruderal plants grow near settlements. All these taxa exist and grow in the region today.^82^

### Morphology and taphonomy of faunal material

**Figure 3:**
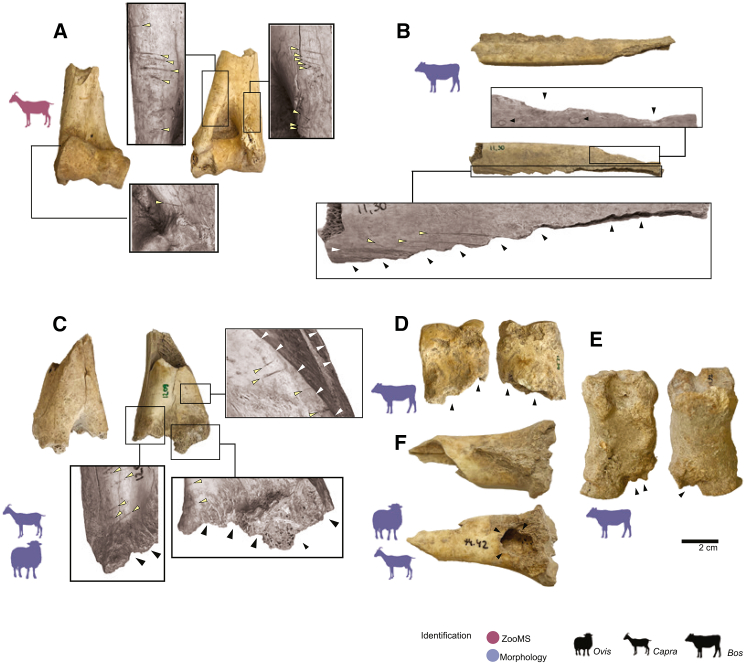
Examples of anthropogenic and carnivore modifications in bone specimens from Yeghegis-1 (A) Goat distal humerus (Sp. 1.305, Horizon 1) with multiple cutmarks on both the anterior and posterior aspects of the medial and lateral epicondylar crests; (B) Cattle rib fragment (Sp. 11.30, Horizon 4), with evidence of carnivore gnawing (black arrows) on both the posterior and anterior borders, as well as multiple scrape (white arrow) and cutmarks (yellow arrows) on the ventral aspect of the shaft fragment; (C) Caprine distal humerus (Sp. 12.09, Horizon 2) with evidence of carnivore gnawing (black arrows), as well as chop marks (white arrows) and cutmarks on the medial and lateral epicondylar crests (yellow arrows); (D and E) examples of carnivore gnawing on cattle phalanges (Sp. 12.30, Horizon 2; Sp. 111.92, Horizon 3); (F) Caprine proximal shaft with carnivore gnawing and tooth pit (Sp. 74.42, Horizon 4).

A total of 10,396 faunal specimens were analyzed (excluding mammalian microfauna, i.e., remains of animals <1 kg). We performed thorough taphonomic analyses of the materials to study the accumulation history of the assemblage. The faunal materials from Yeghegis-1 exhibited a high degree of fragmentation, with 87.1% of the skeletal fragments analyzed preserving less than a quarter of the bone’s original circumference and length. Fragments measuring <2 cm dominated the assemblage (77.2%; Figure S3). The assemblage is well preserved in terms of bone surface modifications, with mid-to-heavy weathering observed in only 1.9% and abrasion in 2.2% of the specimens (Figures S4A and S4B). Burning was recorded in 10.8% of the bone fragments studied (Figure S4C) and anthropogenic bone surface modifications (including cut marks, chop marks, etc.; Figure 3) were recorded in 0.6% of the specimens studied. At least 2.2% of the bone fragments exhibited evidence of carnivore modifications (gnawing, tooth pits, etc.; Figure 3), which we hypothesize to have been caused by canids (i.e., dogs, wolves or jackals; see details in supplemental information).

**Figure 4:**
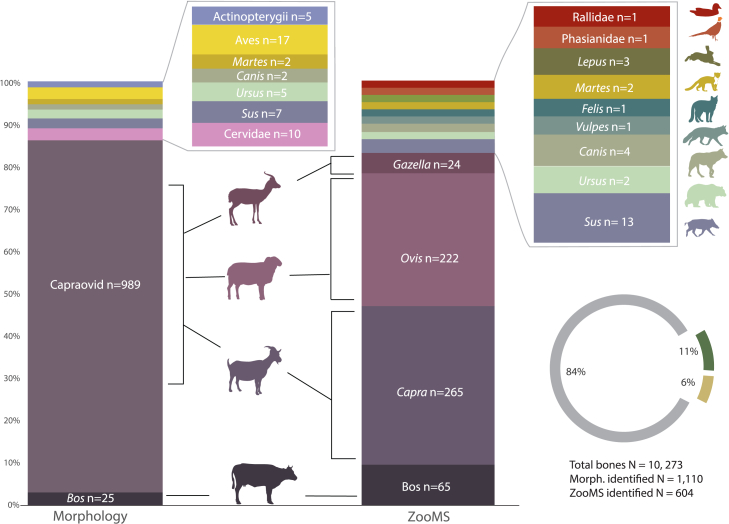
Faunal composition at the site reflecting relative frequencies (%NISP) of taxa identified using morphology and ZooMS See the NISP counts in Tables S4 and S7.

The high level of bone fragmentation severely limited taxonomic identification, with only 10.2% displaying diagnostic morphological features. Morphological identifications revealed the presence of nine taxa. In all Horizons, bovids dominate the assemblage, specifically Caprines (sheep and/or goat: *Capra hircus*), representing 93.4% of the total number of identified specimens (NISP) followed by cattle representing 2.4% of the NISP. Remains of cattle (*Bos taurus*) were also recorded in all Horizons, albeit in very low frequency (*N* = 25; 2.4%). Even though we did not identify the wild counterparts of these ungulates (*Ovis orientalis gmelini*, *Capra aegagrus*, and *Bos primigenius*) in the identifiable portion of the assemblage we do not exclude their possible presence, as these species are known to have been present in the Chalcolithic Caucasus. For instance, in the nearby Areni cave large log size index values are suggested to hint at the presence of very large, possibly wild individuals, counting for only 1.9% of total NISP at the site (*n* = 39).^83^ Other ungulates are represented by cervid/antelope, (*N* = 10; 0.9%) and boar (*N* = 7; 0.7%) remains are scarce. The morphological screening revealed one specimen of hare in Horizon 4. A rare occurrence of carnivores was recorded, represented by *Ursus* (*N* = 5), *Canis* (*N* = 1), *Vulpes* (*N* = 1), and *Martes* (*N* = 2). Additionally, 17 bird specimens were identified (Figure 4; Table S4; Figure S5).

Age-at-death data from dental eruption sequence, dental wear, and appendicular skeleton fusion stages indicate the presence of both young and adult Caprines in the assemblage, with a slight predominance of animals aged 2.5 years or younger (Figure S7). See further details in supplemental information.

### Zooarchaeology by mass spectrometry

To complement morphological identifications and refine the taxonomic composition of ungulates, including species that are difficult to distinguish morphologically, and to explore the potential for identifying human remains, we applied zooarchaeology by mass spectrometry (ZooMS) to 640 bone fragments recovered from all layers of the site. In particular, 640 remains were randomly selected (in order to try to produce data representative of the diversity of fragment sizes and anatomical parts found at the site, from each Horizon and Subhorizon of the trench, coupled with all dental elements preserving roots. The selection of samples was guided by the number of fragments per horizon, the available resources, and the objective of obtaining a representative dataset that accurately reflects the overall diversity of the assemblage. Morphological screening of the assemblage revealed four main categories of remains based on anatomical classification: unidentifiable small fragments (<2 mm), unidentifiable long bone fragments, unidentifiable flat bone fragments, and anatomically identifiable specimens. To ensure that the ZooMS dataset adequately represents the assemblage’s diversity, we aimed to randomly select an approximately equal number of bones (*n* = 16–19) from each category within each Horizon and Subhorizon of the site. This approach facilitated an even distribution of ZooMS samples across the archaeological strata. The inclusion of <2 cm fragments allowed for the identification of smaller taxa. During the selection process, burnt and heavily abraded bones were excluded to optimize collagen preservation and maximize taxonomic identification success. Additionally, all teeth that preserved roots were also selected for ZooMS identification (Table S5).

ZooMS of the 640 bone fragments provided additional taxonomic identifications for 604 specimens (i.e., 94% retained sufficient collagen for identification). ZooMS screening revealed the presence of 13 taxa, with 98% identified to genus level (Figure 4; Table S7; Figure S8). Sheep and goats dominate the record (80% of ZooMS identified fauna). The second dominant group is cattle (10%), noticeably more frequent than in the morphological assemblage. The higher representation of *Bos* sp. in the ZooMS dataset is not surprising, as ZooMS counts often correlate with taxon body size in highly fragmented assemblages, where larger bones break into a higher number of fragments.^84^^,^^85^ This tendency of ZooMS to inflate NISP has been a recurring topic of discussion, with various approaches being proposed to improve the quantitative integration of ZooMS with zooarchaeological datasets.^84^^,^^85^^,^^86^^,^^87^^,^^88^ Additionally, ZooMS revealed the presence of gazelles, which were not identified morphologically, due to their close resemblance to Caprines, especially in the postcranial elements. A broad range of carnivore taxa was also recorded, including mustelids (*Martes* sp.), canids (*Canis* sp. and *Vulpes* sp.), felids (*Felis* sp.), and ursids (*Ursus* sp.), in limited numbers (≤3%). The small non-mammal component of the assemblage (2%) is represented by birds (Rallidae and Phasianidae families; See further details in supplemental information).

All specimens identified by ZooMS were grouped by morphologically assigned body size classes (Figure S9) and bone cortical bone thickness values (Figure S10A) to refine previous morphological estimates. To evaluate the efficiency of the sampling strategy and its possible impact on the obtained results, we grouped the taxa identified through ZooMS into four categories: unidentifiable small bone fragments (<2 mm), unidentifiable long bone fragments, unidentifiable flat bone fragments, and anatomically identifiable specimens (Figure S10B).

### Collagen stable isotope analysis of herd animals

Collagen stable isotope analysis was utilized to explore the long-term dietary habits and ecological conditions of Caprines, offering insights into potential variations in their herding environments and management practices. Twelve Caprines were screened using collagen stable isotope analysis (Table S8). C:N ratios ranged between 3.2 and 3.6 indicating good preservation for all specimens (1.0‰ tolerance)^89^^,Table 4^. The collagen carbon and nitrogen content ranged from 39.0% to 43.8% and 12.6%–14.8%, respectively (Table S9). Caprines show a range of δ^13^C values from −20.8‰ to −19.0‰, with an average of 19.7 ± 0.6‰ and an overall range of 1.8‰. δ^15^*N* values range from 2.9‰ to 10.9‰, with an average of 6.3 ± 2.5‰ and an overall range of 8‰. δ^13^C bone collagen values suggest a pure C_3_ diet for all Caprines.^90^^,^^91^ Typical δ^15^*N* values reported for terrestrial herbivores in Europe are between 2.2‰ and 6.5‰.^92^ Yeghegis-1 δ^15^*N* values fall within the upper limit of these expected ranges but exhibit an untypically large spread. This variability may reflect synchronic or diachronic differences in herding practices^93^^,^^94^^,^^95^ of Caprines and/or changing environmental conditions.^96^^,^^97^^,^^98^ Statistical analysis (Supplementary Section: Bulk Collagen Stable Isotope Analysis) indicates that while δ^13^C values remain relatively consistent across stratigraphic Horizons, δ^15^*N* values are significantly different, suggesting a diachronic increase from Horizon 0 to Horizon 5 (Figures S20 and S21). The large spread of δ^15^*N* values could therefore be attributed to a diachronic shift in herding, landscape management strategies, or by other evolving environmental or anthropogenic factors.

### Bulk tooth enamel stable isotope analysis of herd animals

Bulk enamel stable isotope analysis was employed to provide insights into the dietary habits and environmental conditions experienced by the herbivores and omnivores, facilitating the reconstruction of past ecosystems, at Yeghegis-1. Tooth enamel specimens representing Bovidae, Cervidae, and Suidae families were sampled (*N* = 41; Table S8) for bulk stable δ^18^O and δ^13^C isotope analyses. Results are summarized in Table S10 and Figure S15. Overall, bulk enamel δ13C values (*N* = 42) range from −12.4‰ to −8.6‰ and bulk δ^18^O values (*N* = 42) range from −8.67‰ to −2.00‰. The range of δ^18^O values falls within the range of variation for a single location reported by other bulk enamel isotope studies.^99^^,^^100^ There is no significant difference in δ^13^C values (ANOVA, *p*-value: 0.1) or δ^18^O values (ANOVA, *p*-value: 0.6) among the Bovidae, Cervidae, and Suidae families, indicating that these taxa likely inhabited and exploited similar water sources and environmental niches.

The Caprines (Ovis/Capra) (*N* = 25) display the greatest variability in δ^18^O values, including the highest values within the assemblage (Figure S17). This may be because Caprines, unlike the other taxa sampled, are non-obligate drinkers. They can also obtain water from sources with higher δ^18^O, such as leaf water, which could result in higher overall δ^18^O values.^80^^,^^101^^,^^102^ In contrast, the two suids sampled fall within the lower range of δO values (Figure S17), likely due to the strictly obligate drinking behavior of pigs.^103^ Differences in values might also be attributed to the tooth sampled, which can, depending on the tooth and wear, reflect attenuated seasonal signals and therefore averages.^104^^,^^105^^,^^106^^,^^107^^,^^108^^,^^109^^,^^110^^,^^111^^,^^112^^,^^113^^,^^114^

The δ^13^C values of almost all herbivores and omnivores analyzed (*N* = 41) indicate a pure C_3_ diet, with the exception of one deer and^18^ one cow which display a small contribution of C_4_ plants (enamel-diet ^13^C-enrichment factor (e∗) of +14.5‰).^115^ None of the specimens display evidence for grazing or feeding in forested environments based on expected values for feeding in more closed settings^116^(Figure 6). Overall, the results highlight the animal that the people of Yeghegis-1 targeted inhabited an almost pure, open C_3_ environment in the Late Chalcolithic. It is worth noting that biome reconstructions for this time period are in line with our results and indicate the presence of a temperate grassland in the region (Figure S22).

**Figure 5:**
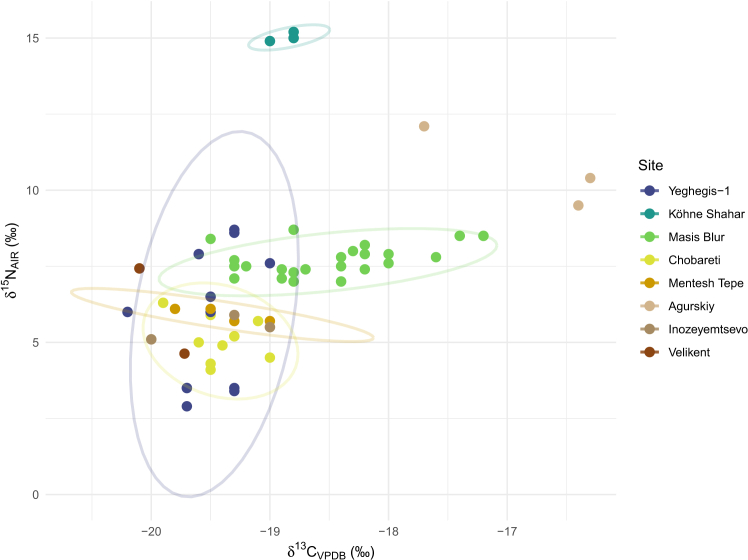
Bone collagen δ^13^C and δ^15^*N* values of sheep and goats from Yeghegis-1 (*N* = 12; this study), Koehne Shahar,^78^ Masis Blur,^74^ Chobareti,^76^ Mentesh Tepe,^75^ Agurskiy,^70^ Inozeyemtsevo^70^ and Velikent^70^ with 95% confidence ellipses for each site Outliers were first removed from the data using the interquartile range (IQR) method, with values outside 1.5 times the IQR from the lower and upper quartiles treated as outliers. This step was performed to ensure that the confidence ellipses for each site were more reliable and not influenced by extreme values. The Yeghegis-1 data are in Table S9 and Figures S18–S20.

**Figure 6:**
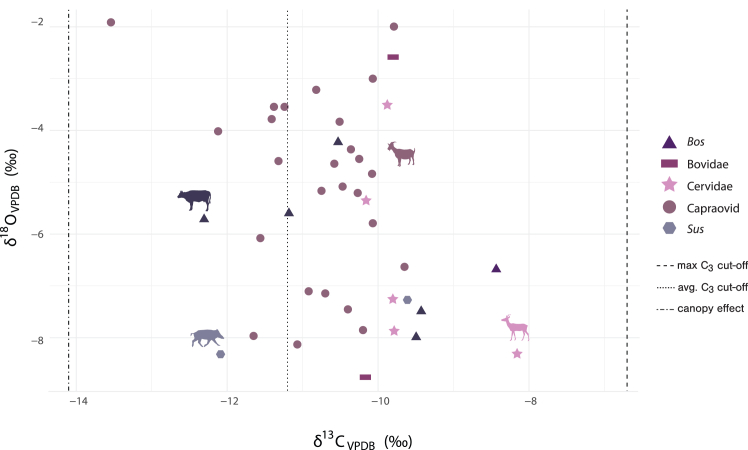
Bulk stable oxygen and carbon isotope analysis of tooth enamel of *Bos* (*N* = 6), Bovidae (*N* = 2), Cervidae (*N* = 5), Capraovid (*N* = 25), and Suidae (*N* = 2) from Yeghegis-1 Maximum C_3_ cut-off is based on local δ^13^C values in archaeological C_3_ plants from Yeghegis-1 (Table S1). The average C_3_ cut-off is based on the global average of δ^13^C values in C_3_ plants.^91^ An enamel-diet ^13^C-enrichment factor (e∗) of +14.1‰ for ruminants was applied.^115^ Data are further summarized in Table S10 and Figure S15.

There is also no significant observable diachronic difference at Yeghegis-1 in δ^18^O and δ^13^C values from bulk enamel between Horizons (see Supplementary section Bulk isotope analysis).

### Sequential tooth enamel stable isotope analysis of herd animals

Sequential stable isotope analysis was utilized to examine the seasonality of husbandry practices, enabling a detailed reconstruction of seasonal behaviors and mobility patterns of the herd animals. A total 124 enamel samples were sequentially collected and analyzed from 14 sheep and goat (taxonomy confirmed via ZooMS) upper and lower molars, recovered from all Horizons. Intra-tooth sequences of δ^18^O and δ^13^C values are shown in Table S11 and Figure 8. Overall, the δ^18^O values vary between −10.2‰ and −0.8‰. In the second molars (M2; *N* = 5) the amplitude of intra-tooth variation is between 4.1‰ and 5.7‰ (when optima are identifiable), while in the third molars (M3; *N* = 6) the amplitude of intra-tooth variation ranges from 3.5‰ to 7.3‰. Specimens Y101.01, Y104.01, Y12.145, Y13.98, Y.32.01, Y43.01, Y6.119, Y76.02, and Y77.01 display a pattern of sinusoidal variation in their δ^18^O values, reflecting an expected seasonal cycle. Specimens Y12.149, Y31.01 and Y4.70 display an attenuated pattern in their δ^18^O values, reflecting incomplete seasonal cycles. This may be attributed to short tooth crowns resulting from tooth wear. Specimen Y.9.43 displays sinusoidal variation with some noise in its δ^18^O values, still reflecting one seasonal cycle. Predicted intra-annual variation in δ^18^O values in modern precipitation at Yeghegis-1 is 9.1‰,^117^ while in general across the Armenian highlands this index has been recorded as 13.7‰.^118^ In all specimens we interpret the minimum δ^18^O values as the colder months and the maximum δ^18^O values as the warmer months (Supplementary Section Stable oxygen isotope analysis). The seasonality in the precipitation is also confirmed by paleoclimatic estimates^119^^,^^120^ (Figure S23).

**Figure 7:**
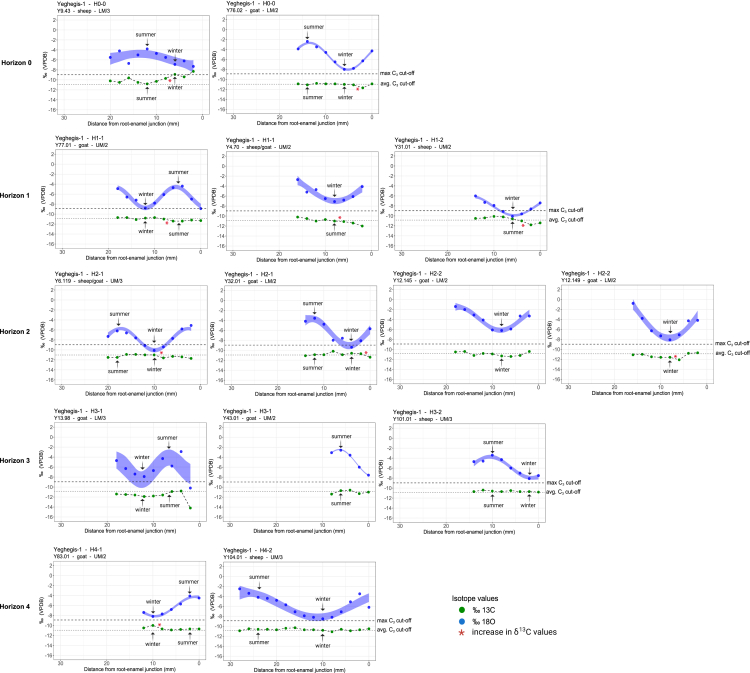
Sequential stable oxygen (δ^18^O) and carbon (δ^13^C) isotope analysis in Caprines (*N* = 14) from Horizons 0–4 at Yeghegis-1 Visualization was performed using the R code published by Hermes et al. (2022).^121^ The maximum C_3_ cut-off is based on local δ^13^C values in archaeological C_3_ plants from Yeghegis-1 (Table S1). The average C_3_ cut-off is based on the global average of δ^13^C values in C_3_ plants.^91^ Data are further summarized in Table S11.

**Figure 8:**
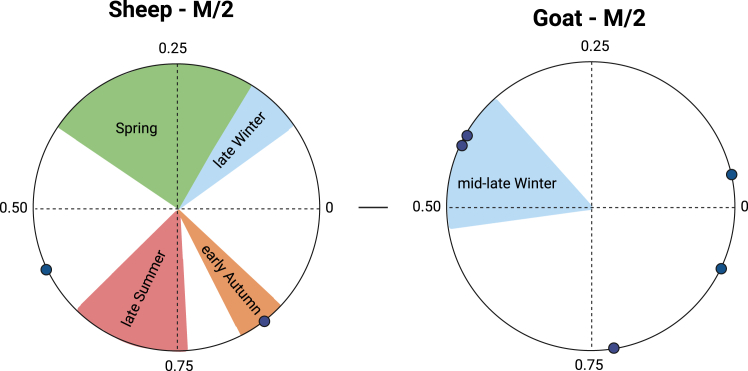
Birth seasons of sheep and goat at Yeghegis-1 are reflected by the position of the maximum δ^18^O value in the tooth crown (x^0^) normalized to the period of the cycle (X) The birth season is compared with modern reference sets. For sheep: Carmejane (CAR),^122^ Rousay (ROU),^123^ Selgua (XT),^124^ Kemenez (KMZ),^125^ North-Ronaldsay (NR),^126^ Le Merle (MRT) and La Fage (MUT)^127^; and for goat: Sagalassos.^128^ Green, blue, orange, and red color areas represent normalized range values obtained from modern specimens. Archaeological specimens are represented in dots. Detailed information about modern sheep and goat reference sets and modeled archaeological specimens are presented in Table S8.

The intra-tooth sequential δ^13^C values display limited variation, with overall values varying between −14.2‰ and −8.3‰ (most of the variation ranges around 1‰ or less. The large overall range is caused by a single specimen Y13.98). In the second molars (*N* = 5) the amplitude of intra-tooth variation is between 0.8‰ and 1.7‰. In the third molars (*N* = 6) the amplitude of intra-tooth variation varies between 0.4‰ and 3.4‰. Stable carbon isotope results are further discussed in the supplementary section stable carbon isotope analysis. The δ^13^C values exhibit minimal variability across all specimens, suggesting a consistent consumption of C_3_ vegetation throughout the annual cycle, where identifiable. Notably, specimens Y9.43, Y76.02, Y77.01, Y4.70, Y31.01, Y6.119, Y32.01, Y12.149, and Y83.01 exhibit a marginal increase in δ^13^C values coinciding with the winter minimum in δ^18^O, as illustrated in Figure 8. Specimen Y13.98 displays an anomalous decrease in δ^13^C values, with the lowest recorded value of −14.2‰. This suggests the potential influence of a canopy effect, indicating that this individual may have been herded through a forested environment during the winter months (Figure 8).

Additionally, we used the δ^18^O sequences to assess animal birth seasons (Figure 8; Table S12). In the upper third molars of sheep (*N* = 2), x0/X ratios vary between 0.12 and 0.64. In the lower second molars of goats (*N* = 3; x0/X) ratios vary between 0.78 and 0.95. In the upper second molars of goats (*N* = 2) the (x0/X) ratios vary between 0.06 and 0.41 (Table S12). These results were compared to modern reference datasets from sheep and goat specimens with a known season of birth (see Table S12 for modern reference sets). Although we are dealing with a limited sample size (*N* = 2), at Yeghegis-1, sheep births seem to have occurred in late summer and late winter. Goat births occurred from summer through to autumn. Both sheep and especially goats exhibit birth seasons that vary throughout the year, potentially indicating an extended birthing period for Caprines (Table S12). However, the interpretation of this dataset requires caution due to its small sample size. Notably, only 6 out of the 14 analyzed specimens were suitable for modeling, which may affect the completeness and reliability of the results. Furthermore, the dataset comprises specimens from different horizons, meaning that the findings do not represent a single, coherent birth pattern but rather a composite of multiple temporal phases. As highlighted by Balasse et al. (2020),^125^ distinguishing individual birth seasons beyond broad seasonal categories becomes increasingly challenging when working with small sample sizes. While such datasets can still reliably differentiate major seasonal trends, more precise conclusions regarding birth distribution should be approached with caution. These limitations highlight the need for expanded datasets to improve resolution and enhance confidence in birth season reconstructions, particularly when attempting to identify finer-scale seasonal patterns.

## Discussion

### Subsistence ecology

Our multiproxy approach to the archaeological assemblage of Yeghegis-1 has yielded direct insights into Chalcolithic subsistence in the Caucasus. Considering the fragmentary nature of the animal assemblage, zooarchaeological analysis faced some challenges especially in the context of identifying closely related species that share almost identical postcranial morphological features (e.g., sheep, goat, and gazelle). Molecular approaches, such as ZooMS, address this limitation by allowing the retrieval of taxonomic information from bone fragments, excluded from morphological zooarchaeological analysis. Therefore, the best results are attained when combining traditional and molecular methods of taxonomic identification. As anticipated, our ZooMS results noticeably increased our understanding of species diversity when considered alongside the morphological results and increased the number of identified species, while being consistent with the data obtained from the morphological analyses. The faunal assemblage, dominated by Caprines throughout the period of site occupation, highlights the importance of these medium ungulates in the subsistence economy of the groups that occupied the site. This pattern is consistent with broader regional trends, where subsistence practices heavily rely on sheep and/or goat herding^21^^,^^24^^,^^41^^,^^44^^,^^51^^,^^60^^,^^67^^,^^83^^,^^129^^,^^130^ and has been suggested as evidence of a specialized and mobile form of pastoralism.^83^ Notably, most regional sites report sheep and goat remains as a broader group of Caprines, thereby limiting the understanding of herd composition. Here, the application of ZooMS allowed us to reveal the herd composition as being composed of both sheep and goats, highlighting the benefits of molecular screening of bones when studying fragmented assemblages. Though the implications of prioritizing mixed herds in Yeghegis-1 are yet to be fully understood, it has been suggested that this practice can stabilize subsistence economies by spreading the risk of failure across species.^83^

Another trait observed at Yeghegis-1 is the limited importance of cattle in the animal economy. While the role of cattle in daily life remains to be fully elucidated, evidence from a contemporary regional site indicates that cattle were used for traction and/or loading.^51^ The presence of wild mammals, along with fish and bird remains, indicates a broader exploitation of natural resources. However, their low frequency suggests that this was likely opportunistic rather than a primary subsistence strategy. This strategy may have been driven by environmental conditions, seasonal availability, or social factors, indicating a complex and dynamic interaction with the surrounding ecosystem. In other regional sites, the presence of wild animals, particularly carnivores, were suggested as being a part of ritual and ceremonial activities, evidenced by specialized burial treatments for these animals.^83^^,^^131^ At Yeghegis-1, however, we did not observe such evidence, suggesting that carnivores were potentially targeted for their fur and/or fat, rather than for ritualistic purposes.

In Yeghegis-1, a small assemblage of cultivated crop remains is accompanied by wild (presumably foraged) berries and weedy or anthropophilic plants which, together with a heavy deposit of ceramic sherds, hearths, and ash layers, suggest the transportation and consumption of plants as well as possible dung burning at the site. Notably, cultivated plants represent only 2% of the total archaeobotanical collection of Yeghegis-1, not including the numerous fragments of Cerealia. While these data are too limited to reconstruct the role of cultivation in the economy at the site, the absence of agricultural tools and plant chaff may suggest a low-investment form of cultivation complementing pastoralism (see discussion in supplemental information: the section on macrobotanical remains). Additionally, the presence of wild berry seeds may represent a component of foraging in the diet. These results are consistent with other regional archaeobotanical data, which point to a focus on wild berry foraging and possible cultivation.^132^^,^^133^ Overall, the results are in line with interpretations of a predominantly pastoral herding strategy adapted to the local environments and opportunistic collection of wild plant and animal resources.^134^

### Herd mobility

The isotope data allowed us to directly assess herd mobility and management strategies at Yeghegis-1. To contextualize our results, we provide an overview of the local environment, hydrology, and vegetation. Yeghegis-1 experiences a continental climate and is surrounded by diverse highland and lowland water sources. The Kura and Aras rivers are the primary waterways, complemented by large freshwater lakes and underlying aquifers that supply groundwater via wells or natural springs.^135^ Snowpacks in alpine and subalpine zones act as natural reservoirs, gradually releasing meltwater.^122^^,^^136^ The predicted intra-annual variation in δ^18^O values in modern precipitation at Yeghegis-1 is 9.1‰, while the broader Armenian highlands show a range of 13.7‰.^91^ Positioned at 1,500 m above sea level, Yeghegis-1 lies in a sub-alpine vegetation zone dominated by C_3_ plants year-round (Figure S22). Surrounding higher elevations feature alpine meadows with a similar C_3_ plant dominance.^124^ Mixed forests in the Lesser and Greater Caucasus mountain ranges contribute to lower δ^13^C values due to the canopy effect.^116^ In contrast, semi-arid and arid steppes in lowland areas such as the Ararat and Sharur plains (40–50 km away) contain C_3_/C_4_ mixed environments in summer and predominantly C_3_ plants in winter.^126^ While C_4_ plants in the Caucasus are summer-adapted, some persist through winter in rocky or sun-exposed areas and are detectable in herbivore diets i.e., in Sharur.^127^^,^^137^ Other lowland plains, such as the Kura-Aras basin, exhibit seasonal shifts between C_3_ and C_4_ vegetation.^138^

In all specimens studied, we interpret minimum δ^18^O values as indicative of colder months and maximum δ^18^O values as reflective of warmer months. Most Yeghegis-1 specimens show a reduced seasonal variation, likely due to higher-than-expected winter δ^18^O values.^90^ This could be linked to the North Atlantic Oscillation (NAO), which influences winter δ^18^O precipitation levels in the Caucasus.^91^ Additionally, winter is not the primary rainfall season in the highlands, with most precipitation occurring in spring and autumn. Caprines at Yeghegis-1 may have obtained much of their winter water intake from leaf moisture, raising minimum δ^18^O values.^80^^,^^101^^,^^102^ Non-obligate drinkers like sheep and goats could also exhibit dampened winter δ^18^O values due to foddering.^27^ Access to isotopically stable spring water^139^^,^^140^ or a milder Chalcolithic climate^141^ could further explain the elevated winter δ^18^O values. Some specimens (6.119, 77.01, 32.01, 83.01) display lower-than-expected summer δ^18^O values, likely due to snowmelt consumption.^140^ Conversely, specimen 12.145 has higher summer values, suggesting reliance on evaporatively enriched leaf water rather than meltwater sources. It should also be noted here that δ^18^O values in the region do not lend themselves to reconstruct altitudinal movements in animals.^118^

Caprines at Yeghegis-1 consumed a pure C_3_ diet with minimal seasonal variation, consistent with the local C_3_-dominant plant community. The δ^13^C values in teeth exhibit limited fluctuation, aligning with typical seasonal changes in C_3_ plants (1‰–2‰), which show higher values in dry summers and lower values in wetter winters.^142^^,^^143^ Some specimens show even lower δ^13^C values (Table S11), suggesting dietary influences such as plant physiology, humidity, temperature, and selective feeding on specific plant parts or foddering.^27^^,^^143^^,^^144^^,^^145^^,^^146^ A slight δ^13^C increase coinciding with winter δ^18^O minima (Figure 7) suggests that herders may have stored and fed C_3_ grasses collected in spring and summer to animals during winter. This increase could reflect the feeding of water-stressed plants, which have higher δ^13^C values,^147^ or consumption of twigs, stems, and roots, which are more accessible in winter and also exhibit enriched δ^13^C values.^148^ Overall, the sequential dataset from Yeghegis-1 supports the interpretation of year-round local herd maintenance.

The isotopic profiles at Yeghegis-1 contrasts with the patterns observed at regional sites where herders practiced transhumance. If the site had been used only as a spring or summer pasture, with herds moving to the lowlands such as the Ararat or Sharur plain in winter, we would expect to see evidence of a C_4_ dietary component and increased variation in δ^13^C values.^74^^,^^127^ Such isotopic signals have been documented in wild sheep at Epigravettian Kalavan-1,^127^ gazelle and domestic Caprines at Neolithic Masis Blur^74^ as well as for sheep at Early Bronze Age Maxta I,^149^ where herds migrated seasonally from lowland plains to highland pastures. At these sites, increased δ^13^C variability reflects the seasonal transition between winter C_4_ components in microenvironments within the plains and summer C_3_ components in highland pastures. In contrast, the late Chalcolithic data from Yeghegis-1 show limited δ^13^C variation and a consistently pure C_3_ diet throughout the year, a pattern similar to findings from Late Bronze Age Tsaghkahovit and Gegharot sites,^64^ located on the slopes of Aragats mountain at a comparable elevation. At these sites, isotopic evidence has also suggested reduced herd mobility.

Broader isotopic studies in the region highlight the diversity of herding strategies used across different periods and sites. Neolithic sites such as Goytepe and Hacı Elamxanlı Tepe in the Kura Basin demonstrate the coexistence of multiple herding systems, including transhumance, year-round lowland pasturing, and seasonal C_3_ foddering.^35^ Later, during the Early Bronze Age in the Kura-Aras Basin, data from Janavartepe indicate a system of year-round pasturing in the plains, where herds alternated seasonally between C_3_ and C_4_ vegetation.^149^ Similarly, isotopic investigations at Early Bronze Age Köhne Shahar in north western Iran reveal limited and short-distance herd mobility, with animals occasionally moved between different highland pastures.^78^ In this broader context, the sequential isotopic dataset from Yeghegis-1 reinforces the interpretation of a localized, year-round herding system with minimal seasonal movement, distinguishing it from transhumant and lowland pasturing practices observed at other sites.

Importantly, our data indicate an absence of lowland use in both winter and summer, suggesting a deliberate year-round highland occupation. Long-distance herd movements would have altered δ^18^O and δ^13^C values, deviating from observed seasonal patterns. If herds migrated to higher-altitude C_3_ pastures in summer, more pronounced intra-individual δ^18^O fluctuations would be expected. However, altitude does not affect δ^18^O values in the Armenian highlands,^118^ meaning that seasonal migration to higher elevations would not necessarily dampen δ^18^O variation. Instead, δ^18^O values could decrease due to snowmelt consumption or increase due to temperature-driven effects on summer precipitation.^150^^,^^151^ Since most precipitation in the Armenian highlands occurs in spring and autumn, when temperatures remain below the 20°C threshold for such effects,^150^^,^^151^^,^^152^ these processes are unlikely to influence the observed isotope data. Additionally, summer precipitation variability, including afternoon thunderstorms,^153^ would complicate interpretations. If herds accessed higher-altitude C_3_ biomes, larger δ^13^C variations would be expected due to altitude effects on plant carbon isotope values. A study near Köhne Shahar found a +0.46‰ δ^13^C increase per 100m of altitude (or +0.36‰ excluding trees), exceeding previous estimates (+0.12‰).^154^ Such movements would produce coinciding peaks in δ^18^O and δ^13^C values, which are absent in Yeghegis-1 specimens, further supporting a localized, year-round herding system. This also strengthens the identification of these specimens as domestic rather than wild.

Although herders and herds may have accessed other C_3_ biomes at similar elevations, such as those in the Lesser Caucasus highlands, this cannot be confirmed without further isotopic analysis. Long-distance movements to the north- and south-east seem unlikely, as traversing forested environments would introduce a detectable canopy effect in δ^13^C values. Future strontium isotope analysis will help confirm specific pasturing locations. No synchronic or diachronic patterns in seasonal herd management were identified, except for specimen Y13.98, which may have passed through a forested environment in winter (Figure 7).

### Herd management

Collagen isotopic data provided further insights into herd management, environmental conditions, and dietary influences on Caprines. Caprine δ^15^*N* values show substantial variation, ranging from 2‰ to 10‰, with a progressive decline from Horizon 5 to Horizon 0. Compared to other sites, the δ^15^*N* values at Yeghegis-1 present a broad distribution. The lowland site of Masis Blur in the Ararat Plain exhibits higher δ^15^*N* values (7.0–11.8‰),^76^ while the highland site of Chobareti,^76^ at a similar elevation to Yeghegis-1, reports values ranging from 4.1‰ to 6.3‰, largely overlapping with those from Yeghegis-1 (Figure 5). These results, alongside stable carbon isotope values confirming a C_3_-based diet, align with expectations for highland environments. Despite the broad spread in δ^15^*N* values at Yeghegis-1, this variation does not necessarily indicate pasturing across both highland and lowland environments. Lowland sites like Masis Blur display elevated δ^15^*N* values alongside carbon isotope data indicative of a mixed C_3_/C_4_ landscape, reflecting the surrounding vegetation. In contrast, the δ^13^C values at Yeghegis-1 remain consistent with a pure C_3_ environment, suggesting that lowland pastures were not utilized by its herders. Instead, the unexpectedly high δ^15^*N* values in some Yeghegis-1 Caprines must be attributed to other factors.

Several explanations would account for this enrichment. The δ^15^*N* values at Yeghegis-1 are comparable to or even exceed those reported for regional carnivores (8.3‰–10.5‰)^74^^,^^75^ and resemble those observed at Mentesh Tepe (300 m a.s.l., Kura Valley), where Caprine δ^15^*N* values range from 5.7‰ to 7.4‰.^75^ Such elevated nitrogen isotope values are often linked to grazing in arid environments or nitrogen-enriched pastures. Two primary mechanisms could explain this pattern at Yeghegis-1: (1) Aridity, which increases δ^15^*N* values in soils and plants through enhanced evaporation and reduced nitrogen turnover,^80^^,^^101^^,^^102^ and (2) Manuring, a process that significantly elevates plant δ^15^N levels when livestock are penned or repeatedly grazed in confined areas.^75^^,^^99^^,^^100^

Increased δ^15^*N* values due to manuring are well-documented, as penning animals concentrates manure, enriching soils and leading to higher plant δ^15^N levels.^155^ A regional comparison further supports this possibility: Caprines from arid steppe sites in the North Caucasus (e.g., Aygurskiy) show enrichment in both carbon and nitrogen isotopes compared to those from more humid forested environments (e.g., Velikent and Inozyemtsevo).^70^ However, at Yeghegis-1, the absence of a significant correlation between carbon and nitrogen isotope values (Spearman’s ρ, *p* = 0.221) suggests that factors beyond diet alone influenced δ^15^N levels. These findings point toward a combination of localized environmental conditions, possible penning practices, and microhabitat variability rather than seasonal movement between lowland and highland pastures.

While our data cannot provide a definitive answer, we propose—drawing on other site-specific bioarchaeological evidence—that elevated nitrogen values in Yeghegis-1 Caprines result from foddering with manured plants or penning.^149^ Penning, a practice for dung accumulation and collection, is further supported by the dominance of weedy plants in the archaeobotanical assemblage. Though some seeds may have entered through seed rain, their concentration likely reflects endozoochory by sheep and goats. Notably, Amaranthaceae seeds, identified as dung-burning indicators at other Eurasian sites,^150^ are common in heavily grazed landscapes.^151^^,^^152^^,^^153^

Additionally, we attempted to reconstruct the organization of the seasonal pastoral cycle and the overall intensity of production and year-round occupation at Yeghegis-1 through the study of livestock birthing seasons. With six specimens, Yeghegis-1 is the only and largest ever published dataset for the Chalcolithic of the Caucasus as a whole. At Yeghegis-1, isolated sheep births occurred in summer and early autumn, while goat births spanned from summer to autumn, extending through much of the annual cycle. These estimates contrast with their wild counterparts (bezoar goat and Armenian mouflon), which primarily give birth in spring.^73^^,^^156^ The extended birthing seasons observed in the dataset could suggest that herders influenced reproductive timing to distribute births more evenly across the year in an attempt to extend the access to milk, meat or fiber. However, we acknowledge that the dataset is not fully representative, limiting the extent to which definitive conclusions can be drawn about broader birthing patterns.^125^ The modest sample size and the presence of specimens from different temporal horizons make it difficult to determine whether this pattern reflects an intentional management strategy, natural environmental influences, individual variability, or sampling biases.

The dataset may contribute to ongoing debates about early selective herd management in the Caucasus, potentially predating previously identified examples from the Late Bronze Age by over 3,000 years.^73^^,^^77^ The presence of all age categories in the Caprine mortality data (Figure S7) suggests a reduced level of herd mobility,^157^^,^^158^^,^^159^ potentially supporting a mixed production strategy incorporating resources such as wool, hair, and milk. The use of dairy products from sheep and cattle is evidenced in proteomic and vessel content analyses from the 5th millennium BCE in both the North and South Caucasus, with an apparent intensification of dairy production among Kura-Araxes herders by the late 4th millennium BCE.^17^^,^^160^ While limited herd mobility and multi-season births are often interrelated, the current dataset does not allow for a definitive assessment of their relationship. Nevertheless, if the observed pattern reflects intentional management, it may indicate early efforts to sustain year-round availability of secondary products, reinforcing the viability of more permanent occupation at the site.

### Reassessing pastoral mobility

The comprehensive archaeobotanical, zooarchaeological and stable isotope analysis for the Chalcolithic at Yeghegis-1 thus reveals a low-mobility, settled system, contrasting with previous models that emphasize specialized forms of mobile pastoralism, such as vertical and horizontal transhumance, in the development of interregional dynamics in Southwest Asia during the 5th millennium BCE. These earlier models proposed that seasonal migrations and extensive trade networks enabled pastoral communities to act as intermediaries, fostering economic integration and cultural interactions, in turn transforming the South Caucasus from an isolated region into a dynamic hub of innovation and connectivity, driven by the activities and exchanges of mobile pastoralists.^21^^,^^24^^,^^25^^,^^41^^,^^42^^,^^48^^,^^49^^,^^50^^,^^129^ However, our findings suggest that mobility among specialized pastoralists may not have been the primary driver for exploiting highland resources or interregional connectivity. It is possible that a diverse range of pastoral strategies existed across the region in the Chalcolithic. Evidence from Yeghegis-1, such as the sourcing of obsidian artifacts,^79^ indicates increased regional exchange during the Late Chalcolithic, despite low physical mobility of the population. This implies that, while the movement of people may have been limited, there was significant intensification in the exchange of goods, knowledge, and raw materials with other regions. Mobile pastoral models have also been challenged by others relating to the trajectory of the Caucasus.^135^

Overall, our results suggest uncertainty regarding existing diachronic models of pastoral mobility for the Chalcolithic Caucasus, which typically correlate the intensification of social networks, low degrees of cereal cultivation, and greater reliance on Caprines as an indicator of increased pastoral mobility. Instead, our findings highlight the complexity of the local and regional dynamics and the need for further studies incorporating a broader range of evidence and proxies to reveal the mechanisms at play. In the Neolithic Zagros, for instance, increased distribution of obsidian among more settled communities was proposed as a result of growing population densities, highlighting intercommunity contacts as a driver for social change.^136^ Another explanation for expanded interregional connectivity could be the presence of specialized trade networks and exchange systems, which may have facilitated the movement of goods, ideas, and technologies across regions, independent of herd mobility. During the Halaf period in the Near East, a network of interconnected, multisite communities with frequent interactions among people has been proposed as a mechanism for fostering connectivity and knowledge transfer.^161^^,^^162^ Our findings underscore the importance of considering multiple developmental pathways for ancient and modern societies, rather than assuming a one-size-fits-all approach to progress and modernization. Future research is needed that incorporates samples from additional regional sites and employs supplementary methods, such as strontium isotope analysis, to provide deeper insights into broader regional dynamics.

### Limitations of the study

This study advances our understanding of human land use strategies in the Chalcolithic Caucasus, integrating multiple lines of evidence to reconstruct human subsistence patterns. However, we acknowledge several limitations of the study. One key limitation is the restricted sample size of the analyzed material. Although archaeobotanical, zooarchaeological, and taphonomic screening was conducted on all recovered bone fragments, only a subset of samples was selected for ZooMS analysis due to resource constraints. Despite this, we believe that the applied sampling strategy effectively captured the faunal diversity at the site, allowing for a representative assessment of the assemblage. Additionally, the targeted ZooMS identification of dental elements selected for stable isotope analysis contributed to refining isotopic interpretations.

While this study contributes to the growing body of isotopic research in the Caucasus, our dataset would benefit from a larger sample size and a greater number of local isotopic studies. While bulk carbon and oxygen isotope analysis was performed on all dental remains, sequential isotope analysis was restricted to specimens that had complete crowns preserved. As a result, the number of teeth available per archaeological horizon was limited, impacting the robustness of seasonality of birth models, which could only be applied to six individuals. Eliminating sequences that cannot be modeled may introduce bias if certain birth seasons are disproportionately affected. This could influence the overall birth seasonality patterns observed in the dataset. Further, current modern reference sets are based on European datasets.^122^^,^^124^^,^^125^^,^^126^^,^^127^^,^^137^ Further, even though our study aligns with other published sequential studies, this approach remains a biographical approach that traces individual life histories, and therefore, given sample constraints, only assesses a small part of the population. This limitation may lead to an overrepresentation of certain life histories while underrepresenting others. Further, our study does not currently include any strontium isotope analysis, which would have provided more specific localities where animals would have grazed and therefore more robustly established animal mobility at the site.

While the multifaceted data from this study raises uncertainty regarding existing diachronic models of pastoral mobility and its role in wider socioeconomic connections for the Chalcolithic Caucasus, we nevertheless acknowledge that this is a perspective from one site. Future research should aim to include additional sites, expand sampling efforts, and incorporate other methods (such as strontium isotope analysis) to further our understanding of past human-earth system interactions in the Caucasus.

## Resource availability

### Lead contact

Further information and requests for resources should be directed to and will be fulfilled by the lead contact, Mariya Antonosyan (antonosyan@gea.mpg.de).

### Materials availability

(1) This study did not generate new unique reagents.
(2) This paper does not report original code. The archaeological materials of Yeghegis-1 rock shelter are stored in the Yeghegnadzor Regional Museum.
(3) The article includes and analyses existing data from previous publications and listed in the key resources table.
(4) All data generated or analyzed during this study are included in this paper and its supplemental information files.

### Data and code availability

• All data generated in this study are available in the Main Text or supplemental information section of this article and listed in the key resources table.
• All ZooMS spectra for identified samples are available on Mendeley at https://doi.org/10.17632/vgy8x5z98v.1 and are publicly available as of the date of publication.
• This paper does not report original code.
• Any additional information required to reanalzse the data reported in this paper is available from the lead contact upon request.

## Acknowledgments

We are grateful to the staff of Yeghegnadzor Regional Museum, Armenia, whose generous support has made the fieldwork possible. We thank Karine Stepanyan, the director of Yeghegnadzor Regional Museum. We are also grateful to Gayane Nazaryan, Roman Nazaryan, Artyom Nazaryan, and Karen Manukyan, as well as local and international volunteers and students who took part in the fieldwork.

The research was funded by the 10.13039/501100004189Max Planck Society. The field work was co-funded by the 10.13039/501100004189Max Planck Society and the Higher Education and Science Committee, MESD, Armenia. L.Y. and S.M. are supported by the Higher Education and Science Committee, MESCS, Armenia (Projects #21AG-1F025 and #23AA-1F020) and the Presidium of the 10.13039/100008721National Academy of Sciences of Armenia. M.A. and S.M. acknowledge funding by 10.13039/501100000348Calouste Gulbenkian Foundation. A.H. acknowledges funding by EU MSCA-IF under Grant Agreement 101063265. R.S. and K.B. acknowledge funding provided by the 10.13039/501100000781European Research Council, grant number 851102, Fruits of Eurasia: Domestication and Dispersal (FEDD). P.R. acknowledges funding provided by the 10.13039/501100000781European Research Council, grant number 850709, PANTROPOCENE.

## Author contributions

Conceptualization: M.A., G.M., and P.R.; project administration: M.A.; excavations: M.A., S.M., M.S., N.A., E.F., and K.A.; investigation: M.A., S.M., and K.B.; software: M.A., G.M., and S.M.; visualization: M.A, G.M., F.S., and N.A.; funding acquisition: M.A., N.A., P.R., and L.Y.; resources: J.I.; writing – original draft: M.A., G.M., K.B., N.A., and A.H.; writing – review and editing: F.S., R.D., A.B., J.I., R.S., E.F., S.R., R.P., and L.Y.

## Declaration of interests

The authors declare no competing interests.

## STAR★Methods

### Key resources table

**Table:** 

REAGENT or RESOURCE	SOURCE	IDENTIFIER
**Biological samples**		

Ancient animal skeletal remains	This study	Figure S3
Ancient plant remains	This study	Figure S2

**Deposited data**		

MADI TOF MS spectra	This study	Mendeley Data https://doi.org/10.17632/vgy8x5z98v.1
Taphonomic characteristics of animal skeletal remains	This study	Figures 3 and S2–S4
Taxonomic identifications of faunal remains		Tables S4, S5, S6, and S7; Figures 4, S5, S6, and S8
Taxonomic identifications of botanical remains	This study	Table S2
Isotope measurements	This study	Tables S9, S10, S11, and S12; Figures 5, 6, 7, 8, and Figures S11–S21
Paleoclimatic dataset	This study	Figures S22 and S23

**Software and algorithms**		

Mmass v5.5.01	Strohalm et al. 2010^142^	https://mmass.findmysoft.com/
R package pastclim v. 2.0.0	Leonardi 2023^119^	https://github.com/EvolEcolGroup/pastclim
δ^18^O sequences modelling	Balasse et al. 2012^131^	Figure 8; Table S12
Bayesian OxCal v4.4 model	Ramsey, 2001^143^	https://c14.arch.ox.ac.uk/oxcal.html

### Experimental model and study participant details

This study reports on the zooarchaeological, taphonomic, palaeoproteomic, stable isotope, radiocarbon and archaeobotanical analysis of archaeological remains. Ancient faunal and botanical remains from Yeghegis-1 rock shelter were retrieved during excavations at the site, led by M. Antonosyan and M. Saribekyan. Necessary permits for excavations were obtained from the Ministry of Education, Science, Culture and Sports of the Republic of Armenia. All necessary permits for analyses were obtained from the Institute of Archaeology and Ethnography, Republic of Armenia and Yeghegnadzor Regional Museum, Republic of Armenia. All archaeological samples were curated in Armenia, in sterile plastic bags and given specimen identifiers. Subsequent analysis of remains sampled from Yeghegis-1 rock shelter were conducted at the Max Planck Institute of Geoanthropology in Jena, Germany.

### Method details

#### The site excavations and the studied material

In 2022, a trench (2 × 2m) situated on a relatively flat area near the entrance of the shelter was excavated to a depth of 2m. All materials were collected and recorded according to the established stratigraphic divisions (Horizons and Subhorizons). Bones and other fossils recovered during excavation were collected *in situ* and their stratigraphic position recorded. Excavated sediments were removed for dry sieving with 2- and 0.5-mm sieves to recover smaller specimens. All finds were brushed and cleaned in a field laboratory and stored in airtight, opaque bags. The stratigraphic sequence of Trench 2 was divided into five Horizons (H1, H2, H3, H4, H5), further subdivided into Subhorizons (H1Sp1, H1Sp2, H2Sp1, H2Sp2, H3Sp1, H3Sp2, H4Sp1, H4Sp2, H5′Sp1, H5Sp1) on the basis of visible differences in the sediment (e.g., colour, texture, and presence of rocks; Figure 2) and in the abundance of cultural materials (e.g., ceramic, lithics, ores, charcoal, and animal bones). Horizon 0 represents the topsoil, while Horizon 5 is the last layer excavated. All layers reveal the presence of charcoals, obsidian, bones, and ceramics, which are evidence of the cultural layers in the site’s stratigraphic sequence.

The 2022 excavations revealed ca. 11,000 bone and 77 tooth specimens, coupled with ca. 2,000 ceramic sherds, ca. 1,000 stone artefacts, ca. 100 pieces of copper slag, and 9 copper artefacts. Additionally, 1,151 charred seeds or large charred seed fragments were recovered, together with additional 3,785 non-mineralized and non-carbonized plant remains. The faunal remains were unevenly distributed between stratigraphic layers (Horizons), with the topsoil (also called Horizon 0) containing the smallest amount of remains and Horizon 2 being the richest. The distribution of recovered faunal remains across stratigraphic layers is displayed in Table S3.

Results of the in-depth zooarchaeological analyses, including comprehensive zooarchaeological and taphonomic analyses, will be detailed in a forthcoming publication. The current study does not include analyses of micromammal remains, such as those of murids and cricetids (N=ca. 700), which we hypothesise to have been living at the site (i.e. no clear indications of human modification). Instead, here we focus on taxonomy and diets of intermediate and large-sized mammals.

#### Radiocarbon dating

The chronology of the site was originally reported by Antonosyan et al. (2024)^81^ and Frahm (2024).^79^ Here, we report dates based on ^14^C determinations of two seed specimens: a wheat grain (from Horizon 2, Subhorizon 2, Flora 6, laboratory code UGAMS-69338) and a lentil seed (from Horizon 2, Subhorizon 2, Flora 6, laboratory code UGAMS-69339). The dating was performed at the Center for Applied Isotope Studies, University of Georgia, USA (Table S1). The analysis returned dates of 3783-3649 and 3659-3626 calibrated BCE, respectively, fitting well within the existing chronology of the site (Figure 2; Table S1). The reported dates were calibrated in OxCal 4.4 using the calibration curve IntCal20.14c.^138^

We revise the chronology of the site based on 15 radiocarbon dates. We model the chronology using Bayesian statistics with a site Sequence, using Transition Boundaries between Phases (Horizons) (see OxCal Model Code in the supplementary materials). To correct small overlaps in calibration (H2 SP1 to H2 SP2) and one inversion on the site chronology (H1 SP1), we employed an Outlier Model to dates indicated with (∗) in the table and the code. The new dates allowed for a higher understanding of the site’s stratigraphy, and the chronological modelling points to a general pattern of short transition periods between occupation layers (av. 130 yr).

The resulting dates range from 4194–4046 cal BCE (Horizon 5, the lowest excavated layer) to 3645–3531 cal BCE (Horizon 0). Altogether, this sequence of dates indicates an occupation at the site from the end of the Middle Chalcolithic (ca. 4100–4000 BCE) through the end of the Late Chalcolithic (ca. 3600–3500 BCE) in this region.

#### Macrobotanical remains

Samples for archaeobotanical analysis were retrieved from all archaeological layers of Yegheris-1. The assemblage consists of 56 samples in total (10 litres each). Each sample was floated in the field using a flotation machine method and divided according to the buoyancy of the material into two sub-samples: heavy fraction ≥2mm and light fractions ≥0.5mm. Following the standard processing procedure, as described by Pearsall (2016)^139^ and Marston et al. (2015).^140^ Both fractions of each sample were packed in a cotton bag and air-dried in the shade. Further, the heavy fraction portions were screened in the field laboratory; bone fragments and other artefacts were removed and passed to appropriate specialists for further analysis. Hand-picked plant material from the heavy fraction along with the light fraction of all samples were sent to the paleoethnobotany laboratory of the Max Planck Institute of Geoanthropology in Jena, Germany, for archaeobotanical analysis. Once in the lab, samples were passed through nested U.S. geological sieves to ease sorting. Material smaller than 0.50 mm was not sorted. Carbonised wood fragments larger than 2.00 mm were counted but not identified. Seeds and seed fragments were separated from all sieve units, and desiccated and charred seeds were systematically collected. Identification keys^82^, seed atlases, and manuals for seed identification^142^^,^^163^ were used to identify plant remains and specify their ecotypes. The identified taxa are presented in Table S2, and photos of key taxa were taken with a Keyence VHX600 and presented in Figure S1.

#### Morphology and taphonomy of faunal material

A total of 10,396 animal bone and tooth fragments were recovered during the 2022 excavations of Yeghegis-1 (Table S3). Of these specimens, only 1,058 (10.2%) can be confidently identified to a taxon (family, genus and/or species). The low NISP count is brought about by the high levels of bone fragmentation resulting from anthropic activities at the site.

All bone fragments were sorted, cleaned to allow for observation of surface modifications, measured using a digital calliper, and identified to the highest possible taxonomic classification using modern comparative materials maintained at the Max Planck Institute of Geoanthropology and alongside reference atlases. All specimens were examined for natural marks – including weathering,^143^^,^^144^ abrasion,^145^ and mineral staining – as well as animal and anthropogenic modifications, such as burning and butchery marks (cut marks, chopmarks, etc.) and evidence for carnivore activities.^146^ The completeness of the skeletal element was recorded as well as the fracture patterning^147^ considering the characteristics of fracture surfaces, their position and orientation. All specimens were classified to size based on live weight following a modification of the criteria established by Thomas (1969)^148^ and Grayson (1984)^149^: small mammals (SM, 1–10 kg); intermediate mammals (IM, 10–50 kg); large mammal class 1 (LM1, 50–100 kg), and large mammal class 2 (LM2, >100 kg). Bone fragments that could not be assigned to a taxon but could be identified to a skeletal element were also assigned to a size class considering the relative size of the element (e.g. cortical bone thickness).

#### Zooarchaeology by mass spectrometry (ZooMS)

ZooMS screening was carried out at the dedicated proteomics laboratory at the Max Planck Institute of Geoanthropology following the acid insoluble protocol.^150^^,^^151^ In brief, this involved acid demineralization of 20-30 mg bone chips, isolation and enzymatic (trypsin) digestion of collagen followed by ZipTip purification of the resulting peptides. Samples were run on a Bruker Autoflex Speed MALDI-TOF mass spectrometer (Bruker Daltonics) to produce spectra/fingerprints for taxonomic identification. Extraction blanks were included throughout all stages to monitor the introduction of potential contamination, the blanks were empty of collagen type I, pointing to the absence of protein contamination in the laboratory. The resulting peptide markers were identified via mMass software (v5.5.01^152^), and the registered collagen fingerprints of each specimen are presented in Table S6.

For taxa that exhibited an identical series of markers, taxonomic identifications were assigned considering the current range of fauna in the study region and the archaeological records from the area. This is the case for many wild bovids and cervids that share many of the same peptide markers. For instance, *Ovis* sp., *Rupicapra* sp., and *Nesotragus* sp. have an identical set of markers; however, considering that *Nesotragus* sp. and *Rupicapra* sp. have not been reported in the study region (i.e. outside the biogeographic range of modern and fossil specimens), the attribution to *Ovis* sp is more probable.

Discriminating gazelles (*Gazella* sp.) from deer (*Cervus* sp.) is less straightforward, as both taxa are common in the region and display a similar set of markers. However, a recent study by Janzen and colleagues (2021)^153^ suggested markers to separate members of Antilopini tribe, these are COL1A2 375 and ɑ2 889 that, for Antilopini, display m/z 1182, 2056, 2072 and 1532, respectively. At the same time, m/z 3227 was suggested as a potential marker for gazelles^154^ while m/z 2216 was reported as being specific to red deer.^153^^,^^164^ We used these findings to guide our identifications, with samples which had at least three of the above-mentioned markers identified as gazelle or deer. If the markers were absent, the identification was restricted to *Gazella/Cervus*. Similarly, sheep and goat exhibit almost identical peptide markers with exception of markers COL1ɑ2 757 (+16; m/z 3017.4, 3033.4 for sheep and m/z 3077.4, 3093.4 for goat: Buckley et al. 2009^150^; 2010^165^) and a recently identified COL1ɑ2 375 (m/z 1154, 2028 and 2044^153^; that facilitate the identification of Caprines.

#### Bulk and sequential stable isotope analysis of tooth enamel

During the excavation of Trench 2, a total of 77 teeth were uncovered. Of these, 55 teeth had preserved enamel and belonged to subadult or adult animals, though only 14 retained their complete crowns. All 55 teeth were analysed in this study (41 bulk isotopic sampling and 14 sequential sampling, Table S8). Bulk enamel samples (N=41) reflect various segments and lengths of annual seasonal cycles.^166^ Bulk enamel samples represent the average isotopic values accumulated throughout the process of tooth formation. This approach allows for comparisons of multi-seasonal isotopic averages between individuals.^167^ However, sampling across different taxa with varying feeding and drinking requirements enables the reconstruction of environmental local conditions.^100^^,^^168^^,^^169^ Our sample size aligns with other studies that applied bulk enamel stable isotope analysis to reconstruct past feeding, habitat and environmental conditions.^170^^,^^171^ We acknowledge that the representativeness of the sample varies across horizons and covers a long stratigraphic sequence, which affects interpretations by limiting the ability to identify consistent patterns over time, and increases the likelihood that observed trends reflect individual variability rather than broader herd management strategies. Enamel surfaces were cleaned using a tungsten drill bit. Bulk enamel samples were taken by abrading the complete length of the buccal surface of the teeth with a diamond-tip drill to ensure a representative sample for the whole axis of enamel mineralization. Where this was not preserved, we sampled the lingual aspect.

For sequential analysis, the sample set consists of 14 sheep and goat teeth from Yeghegis-1 site, generating in total 124 measurements. Species identification has been done using ZooMS. Where this was not possible, specimens are indicated as sheep/goat. One second (M2) or one third molar (M3) was taken from each individual (Table S11). Sequential sampling of enamel was conducted by drilling along the tooth crown growth axis.^172^ In sheep, the crown of the M2 begins formation during the first - second month and is completed at 12 months.^109^^,^^110^^,^^111^ In goats, the crown of the M2 begins formation during the first - second month and is completed at 10 to 13 months.^112^^,^^113^ M2 molars from a given sheep or goat therefore provide a record of approximately the first year of life of the specimen. In sheep, the crown of the M3 begins formation during the 9th-10th month and is completed at 20-22 months.^109^^,^^111^ In goats the crown of the M3 begins formation during the 9th-10th month and is completed at 20-30 months.^114^ M3 molars from a given sheep or goat therefore provide an approximate record of the second to third year of the animal’s life. Enamel surfaces were cleaned using a tungsten drill bit. Sequential samples were taken by drilling with a diamond burr on the buccal side of the teeth, perpendicularly to the crown growth axis.

For both sequential and bulk samples, we followed the standard protocol used at the Stable Isotope Laboratory of the Max Planck Institute of Geoanthropology for the analyses of δ^13^C and δ^18^O from the carbonate portion of tooth enamel bioapatite. Pre-treatment was carried out following an established protocol (adapted from Lee-Thorp et al. 2012^173^; Sponheimer et al. 2005^174^; Ventresca-Miller et al. 2018^175^). The isotope analyses were conducted on a Thermo Gas Bench 2 connected to a Thermo Delta V Advantage Mass Spectrometer. Results are reported as delta (δ) values as parts per thousand (per mil, ‰) difference relative to the international standard, Vienna Pee Dee Belemnite (VPDB), where δ (‰) = ((R_sample_/R_standard_)-1 x 1000 and R is the ^13^C/^12^C or ^18^O/^16^O ratio. The resulting values were compared against International Standards (IAEA-603, IAEA-CO-8, IAEA NBS 18) using a three-point calibration. USGS44 was run as an internal standard. An equid enamel standard was run to assess systematic error (accuracy). Accuracy or systematic error ((u(bias))) was determined to be 0.27‰ for δ^13^C and 0.29‰ δ^18^O on the basis of the difference between the observed and known values of the check standards and the long-term standard deviations of these check standards. Precision (u(Rw)) was determined to be 0.15‰ for δ^13^C and 0.19‰ for δ^18^O on the basis of repeated measurements of calibration standards, check standards, and sample replicates. Using the equations provided by Szpak et al. in 2017,^176^ the total analytical uncertainty was estimated to be ±0.3‰ for both δ^13^C and δ^18^O (see Table S13).

#### Collagen isotope analysis

All bone samples were prepared following the modified collagen extraction method of Longin (1971)^177^ and Dunbar (2016),^161^ see supplemental information for further details). The δ^13^C and δ^15^N ratios of the bone collagen were determined using a Thermo Scientific Delta V Advantage continuous-flow isotope ratio mass spectrometer (CF-IRMS) coupled via a Thermo Scientific Conflo IV to a Costech ECS 4010 elemental analyser (EA) fitted with a pneumatic autosampler at the e Scottish Universities Environmental Research Centre (SUERC) Radiocarbon Dating Laboratory. For every 10 unknown samples, in-house gelatine standards, which are calibrated to the international reference materials USGS40, USGS41, IAEA-CH-6, USGS25, IAEA-N-1, and IAEA-N-2, are run in duplicate. Supplementary analyses of in-house mammoth bone background sample and our in-house standard bone are also routinely measured to check the consistency of the bone collagen separation chemistry. Stable isotope ratios of bone collagen are reported as delta (δ) values relative to an international standard: for carbon isotope ratios in collagen this is Vienna Pee Dee Belemnite (VPDB), and for nitrogen isotope ratios in collagen this is AIR, with precisions of ±0.2‰ and ±0.3‰ for δ^13^C and δ^15^N, respectively. Values are reported using the (‰) notation, where δ (‰) = (Rsample/Rstandard) − 1, and R is the ^13^C/^12^C or ^15^N/^14^N ratio.

In total twelve specimens were sampled, and yielded C:N ratios between 3.20 and 3.62, within the acceptable range of ancient mammal bone collagen (1.0 ‰ tolerance)^89^ Table 4. The decision to analyse twelve specimens was based on comparable sample sizes, ranging between 4 - 21 specimens, in other regional Caprine collagen stable isotope studies, which have successfully used similar sample sizes to infer Caprine herd management practice.^74^^,^^76^^,^^78^^,^^162^

#### Modelling of δ^18^O sequences

The δ^18^O sequences were modelled using an equation derived from a cosine function described in Balasse et al. (2012).^123^

$$\delta^{18}O_{m}=A\cdot \cos\left(2\;\Pi \cdot \left(x-{\;x}_{0}\right)/X\right))+M$$

where δ^18^O_m_ is the modelled δ^18^O; x is the distance from the enamel-root junction; X is the period (in mm), or the length of tooth crown potentially formed over a whole annual cycle; A is the amplitude (=max–min/2; in ‰); x_0_ is the delay (mm); δ^18^O attains maximum value when x=x0; M is the mean (=(max+min)/2) expressed in ‰. Specimens were not modelled that have sequences with a very low amplitude of variation, the absence of a sinusoidal pattern of variation, the absence of a clear maximum or a missing Enamel-Root-Junction (ERJ). Further we rejected modelled results with a period X below 16.0. We analysed six specimens to investigate the seasonality of birth in Caprines. While the sample size is modest, it aligns with other regional studies, such as Chazin (2021),^77^ which used 16 specimens from Gegharot and 8 from Tsaghkahovit, and forthcoming research on Maxta^178^ that examines the seasonality of birth of Caprines using six specimens. Similarly, Hermes et al. (2022)^121^ and Knockaert (2018)^179^ each used six teeth to assess the seasonality of birth of Caprines. These studies demonstrate that meaningful conclusions can be drawn from similar sample sizes within the context of the Caucasus. However, eliminating sequences that cannot be modelled may introduce bias if certain birth seasons are disproportionately affected. This could influence the overall birth seasonality patterns observed in the dataset.

The variability in tooth size is eliminated through normalisation of distances using the period X of the δ^18^O cycle. The position of the maximum δ^18^O values in the tooth crown is therefore expressed as x_0_/X. Results from the modelling of the δ^18^O sequences measured in Yeghegis-1 upper and lower M2s are shown in Table S12. The resulting x_0_/X ratio for each specimen is a reference value for the specimen’s season of birth. Season of birth is estimated by comparison with reference ×_0_/X ratios obtained in modern sheep and goats.^122^^,^^124^^,^^125^^,^^126^^,^^127^^,^^137^ Current modern reference sets are biased towards Europe, highlighting the need for modern reference sets from West Asia. Further, the absence of comprehensive reference data for goat births outside the mid to late winter period hampers the estimation of seasonality of birth. This gap in data makes it challenging to accurately estimate the exact seasons births occur, that deviate from, or do not align directly or opposite to, the existing reference periods. In our study we modelled upper and lower M2s and M3s of sheep and goats. Previous studies have shown that there can be a moderate shift (around 1 month) in the isotopic record between the upper and lower M3s of sheep.^125^ There we corrected the x_0_/X ratios of upper sheep M3s accordingly (+0.073). Currently there are no such studies on goats as well as on isotopic shifts between upper and lower M2s. We therefore did not correct these in our data. All calculations have been carried out using Microsoft Excel. The fitting of the model to the dataset is estimated using the Pearson correlation coefficient (*r*). We consider that the model adequately describes the dataset when *r* ≥ 0.92. All results are shown using a circular representation to reflect the cyclical nature of seasonality (Figure 8) as well as in Table S12.

#### Threshold δ^13^C values in tooth enamel and bone collagen

To estimate the proportion of C_3_ and C_4_ plants in the diet of the specimens, the average δ^13^C value for archaeological plants; wheat (-24‰) and lentil (-22.8‰) recovered from Yeghegis-1 was used (Table S1), which is -23.4‰. Other sites in the South Caucasus have reported δ^13^C values of archaeological barley are reported as -20.8‰ from Chalcolithic Mentesh Tepe (360m)^128^ and -20.5‰ from the highland (1900m) Early Bronze Age site of Chobareti.^76^ Modern C_3_ plants rarely exceed values over -23‰ except in extremely arid regions,^89^^,^^180^ highlighting the benefit of using local plant baselines. We thereby use -22.8% (archaeological plants) as the maximum cut-off value for C_3_ vegetation at Yeghegis-1. The average cut-off value for C_3_ vegetation at Yeghegis-1 is based on the global average modern δ^13^C value of -26.0‰ for C_3_ plants.^91^

Modern plant δ^13^C values were corrected by + 1.5‰ to compensate for the fossil fuel effect. In order to estimate δ^13^C values in enamel, an enamel-diet ^13^C-enrichment factor (e∗) of +14.1‰ for ruminants (sheep/goats in our study) was applied.^115^ Thereby -10.7‰ indicates 100% C_3_ diet based on average δ^13^C values and -9.0‰ indicates 100% C_3_ diet based on maximum δ^13^C values. In temperate environments, low δ^13^C values may be attributed to the canopy effect, i.e., consumption of vegetation grown in a dense forest. We use δ^13^C values of -29.7‰ (modern) or -28.2‰ (archaeological) in plants of the canopy effect, resulting in a δ^13^C value of -14.5‰ in ruminant archaeological enamel.^181^ Enamel-diet ^13^C-enrichment factors (e∗) for suids range from +13.3‰^115^ to +14.6‰.^182^ For cattle and cervids an enamel-diet ^13^C-enrichment factor (e∗) of +14.5‰ has been reported.^115^

The δ^13^C values found in bone collagen typically exceed those in plants by 5%. This can fluctuate because the carbon in collagen primarily originates from the protein in an animal’s diet.^183^^,^^184^ With previously established C_3_ plant δ^13^C values, we expect ∼ -19.5‰ for a 100% C_3_ diet based on average δ^13^C values and ∼ -17.8‰ for 100% C_3_ diet, based on maximum δ^13^C values, in herbivore bone. Anything above this value would indicate a C_4_ component to the diet.

#### Palaeoclimatic dataset

We used the R package Pastclim v. 2.0.0^119^ to assess and extract paleoclimatic values, such as precipitation seasonality and biome distribution. The high-resolution dataset used covers the whole world and consists of a time series of 1000-years intervals up to the last 120,000 years.^120^

### Quantification and statistical analysis

A chi-square test was conducted using R version 4.2.1. to examine variations in Caprine remains across six stratigraphic horizons. The analysis compares the observed counts of Caprines per horizon to expected counts, under the null hypothesis of uniform distribution. Detailed description of the obtained results is presented in the supplemental information.

All statistical analyses of stable isotope datasets were conducted using R version 4.2.1. Statistical significance was defined as *p* < 0.05. Detailed descriptions of the statistical methods and corresponding visualisations for the stable isotope datasets are provided in the Figures S11–S20 and figure legends.

For the sequential stable isotope dataset, comparisons of mean δ^18^O values between stratigraphic Horizons 0 through 4 were performed using both one-way ANOVA and the Kruskal-Wallis test. These tests revealed no statistically significant differences between groups (ANOVA: *p* = 0.79; Kruskal-Wallis: *p* = 0.38). For this analysis, sheep, goat, and sheep/goat specimens were combined (N=11, where N represents the number of sequences), and specimens lacking sinusoidal variation were excluded (Figure S11).

For the bulk stable isotope dataset, δ^13^C values (N=41; N represents individual bulk measurements) across Horizons 0–5 were first evaluated for parametric test assumptions (Figure S13). The Shapiro-Wilk test indicated no significant deviation from normality (*p* = 0.198), and Levene’s test confirmed homogeneity of variances (*p* = 0.140). Based on these results, a one-way ANOVA was applied, revealing no statistically significant differences in δ^13^C values between horizons (*p* = 0.332).

To compare bulk δ^18^O values (N=41; N represents individual bulk measurements) across stratigraphic Horizons 0–5, the dataset was similarly evaluated for suitability for parametric testing (Figure S14). The Shapiro-Wilk test showed no significant deviation from normality (*p* = 0.096), and Levene’s test confirmed homogeneity of variances (*p* = 0.278). A one-way ANOVA was subsequently performed, yielding a *p*-value of 0.060. Although not statistically significant, this result suggests a potential trend in δ^18^O values across horizons.

For the bulk collagen stable isotope analysis, the distributional assumptions of the δ^13^C (N=13) and δ^15^N (N=13) datasets (where n represents individual bulk measurements) were assessed using both graphical and statistical methods (Figures S18 and S19). Histograms and Q-Q plots were generated to visually inspect the data distributions, and the Shapiro-Wilk test was used for formal evaluation. The δ^15^N values showed no significant deviation from normality (*p* = 0.375), while the δ^13^C values deviated significantly (*p* = 0.044), justifying the use of non-parametric testing for δ^13^C.

Given the non-normal distribution and small sample size, the Kruskal-Wallis test was used to compare δ^13^C values across stratigraphic Horizons 0–5. This test revealed no statistically significant differences (*p* = 0.230).

In contrast, δ^15^N values satisfied assumptions for parametric analysis. Levene’s test confirmed homogeneity of variances (*p* = 0.977), and normality was supported by the Shapiro-Wilk test (*p* = 0.375). A one-way ANOVA was conducted to assess δ^15^N variation across horizons (H0, H1 SP1, H2 SP1, H2 SP2, H3 SP1, H3 SP2, H4 SP1, H4 SP2, H5 SP1), revealing a statistically significant difference among group means (*p* = 0.0067).

## Supplemental information

Document S1. Figure S1–S23 and Tables S1, S3, S5, and S8

Table S2. Carbonized and mineralized plant remains recovered from Yeghegis-1

Table S4. Number of Identified Specimens (NISP) of different animal taxa (excluding micromammals)

Table S6. List of recorded ZooMS markers per specimen

Table S7. Number of Identified Specimens (NISP) of animal taxa identified via ZooMS

Table S9. Collagen δ^13^C and δ^15^N isotope measurements

Table S10. Bulk enamel δ^13^C and δ^18^O isotope measurements

Table S11. Sequential enamel δ^13^C and δ^18^O isotope measurements

Table S12. Results from the modeling of the δ^18^O sequences

Table S13. Measurement of standard uncertainty data run with stable isotope samples
