# Supplementary material for: A biomolecular perspective on mobile pastoralism and its role in wider socioeconomic connections in the Chalcolithic South Caucasus
Source: iScience. 2025 May 2;28(6):112544. doi: 10.1016/j.isci.2025.112544 (PMC12148388; doi:10.1016/j.isci.2025.112544)
Supplement: Document S1. Figure S1–S23 and Tables S1, S3, S5, and S8 [file mmc1.pdf]

iScience, Volume *28*

Supplemental information

A biomolecular perspective on mobile pastoralism and its role in wider socioeconomic connections in the Chalcolithic South Caucasus

Mariya Antonosyan, Gwendoline Maurer, Satenik Mkrtchyan, Kseniia Boxleitner, Mariam Saribekyan, Anahit Hovhannisyan, Laura Furquim, Freg Stokes, Ruben Davtyan, Arsen Bobokhyan, Karen Azatyan, Jana Ilgner, Sabine Reinhold, Ellery Frahm, Robert Spengler, Patrick Roberts, Noel Amano, and Levon Yepiskoposyan

1. **Choronology**

Table S1: Radiocarbon dates (all from the 2021–2023 Trench 2 excavations and using collagen extraction) calibrated using IntCal20.14c1. Related to Figure 2. *Samples UGAMS-69338 and *UGAMS 69339 this study

Horizon	Sub-horizon	Reporting No.	Specimen	d13C	d15N	C/N	Reported Age	Calibrated BCE (95.4%)	Modelled (95.4%)
Horizon 0	-	SUERC-123532	phalanX	-19.7	2.9	3.4	4813 ± 24 BP	3589 – 3528	3645-3531
Horizon 1	H1 S1	SUERC-123533(*)	radius	-19.7	3.5	3.2	5229 ± 24 BP	4056 – 3971	3696-3562
	H1 S2	SUERC-108138	occipital	-19.5	6.5	3.6	4917 ± 27 BP	3715 – 3641	3715-3641
Horizon 2	H2 S1	SUERC-123534(*)	tibia	-19.5	6	3.3	4998 + 24 BP	3806 – 3704	3757-3656
	H2 S2	SUERC-108142	occipital	-20.8	6.7	3.6	5000 ± 27 BP	3810 – 3702	3787-3699
	H2 S2	*UGAMS-69338	seed	-24			4950 ± 30 BP	3,783-3,649	3777-3680
	H2 S2	*UGAMS 69339	seed	-22.79			4860 ± 30 BP	3,659-3,626	3775-3676
Horizon 3	H3 S1	SUERC-108144	sesamoid	-19.3	3.5	3.5	5008 + 27 BP	3814 – 3704	3895-3714
	H3 S1	SUERC-105538	phalanx	-19.3	3.4	3.3	5008 + 27 BP	3814 – 3704	3895-3714
	H3 S2	SUERC-108143	phalanax	-20.2	6	3.5	5050 + 27 BP	3952 – 3778	3940-3785
Horizon 4	H4 S1	SUERC-108146	occipital	-19.6	7.9	3.5	5127 + 27 BP	3986 – 3914	3976-3811
	H4 S2	SUERC-108145	occipital	-20.7	10.9	3.6	5138 + 27 BP	3991 – 3934	4037-3849
Horizon 5	H5 S1	SUERC-108147	occipital	-19	7.6	3.3	5190 + 27 BP	4047 – 3958	4052-3968
	H5 S1	SUERC-108148	occipital	-19.3	8.7	3.5	5332 + 27 BP	4142 – 4051	4194-4046
	H5 S1	SUERC-105539	phalanax	-19.3	8.6	3.3	5354 + 27 BP	4136 – 4054	4192-4048

Data S1: OxCal code associated with Bayesian model, related to Figure 2

```
Plot()
{
Curve('IntCal20','IntCal20.14c');
Outlier_Model("General", T(5), U(0,4), "t");
Sequence('Yeghegis1')
{
Boundary('Start H5');
Phase('H5')
{
R_Date('SUERC- 105539', 5354, 27);
R_Date('SUERC- 108148', 5332, 27);
R_Date('SUERC-108147', 5190, 27);
};
Boundary('Transition H5 to H4 SP2');
Phase('H4 SP2')
{
R_Date('SUERC-108145', 5138, 27);
};
Boundary('Transition from H4 SP2 to H4 SP1');
Phase('H4 SP1')
{
R_Date('SUERC-108146', 5127, 27);
};
Boundary(‘Transition from H4 SP1 to H3 SP2’);
Phase(‘H3 SP2’)
{
R_Date('SUERC-108143', 5050, 27);
};
Boundary('Transition from H3 SP2 to H3 SP1');
Phase('H3 SP1')
{
R_Date('SUERC-105538', 5008, 27);
R_Date('SUERC-108144', 5008, 27);
};
Boundary('Transition from H3 SP1 to H2 SP2');
Phase('H2 SP2')
{
```

```
R_Date('SUERC-108142', 5000, 27);
R_Date('UGAMS-69338', 4950, 30);
R_Date('UGAMS-69339', 4860, 30);
};
Boundary('Transition from H2 SP2 to H2 SP1');
Phase('H2 SP1')
{
R_Date('SUERC-123534', 4998, 24){Outlier("General",0.05)};
};
Boundary('Transition from H2 SP1 to H1 SP2');
Phase('H1 SP2')
{
R_Date('SUERC-108138', 4917, 27){Outlier("General",0.05)};
};
Boundary('Transition from H1 SP2 to H1 SP1');
Phase('H1 SP1')
{
R_Date('SUERC-123533', 5229, 24){Outlier("General",0.05)};
};
Boundary('Transition from H1 SP1 to H0');
Phase('H0')
{
R_Date('SUERC-123532', 4813, 24);
};
Boundary('End Phase H0');
};
};
```

2. Macrobotanical remains

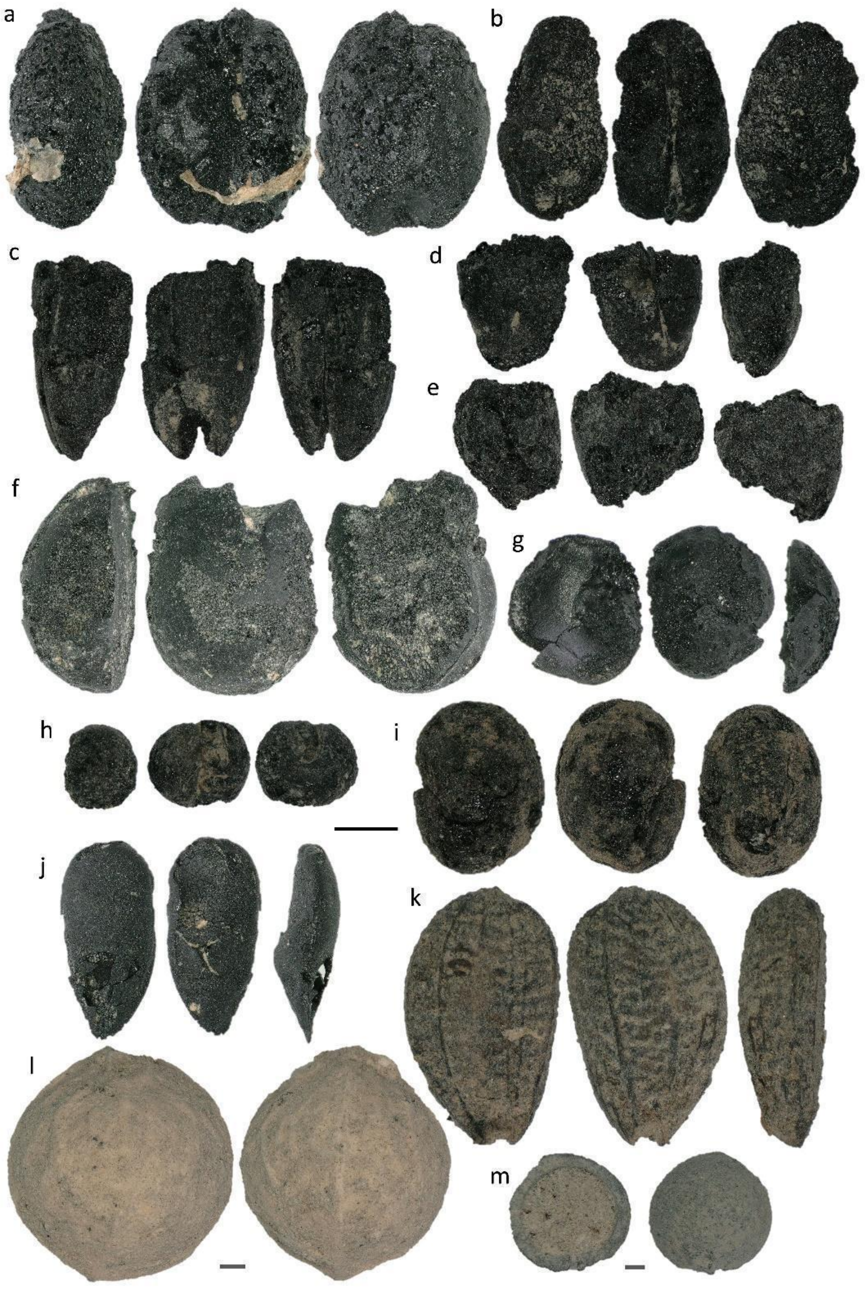

Figure S1: **Selected charred cereal grains, pulse seeds, weedy seeds and a mineralised stone of hackberry from Yegheris-1 rock shelter**: a. free-threshing wheat (*Triticum aestivum*) sent to AMS dating; b. *Triticum aestivum*; c. *Hordeum vulgare*; d-e. Cerealia; f. cf. *Cicer arietinum*; g. a lentil (*Lens culinaris*) cotyledon sent to AMS dating; h. *Vaccaria hispanica*; i. Fabaceae, cf. *Medicago/Melilotus* spp.; j. cf. *Linum usitatissimum*; k. *Onopordum acanthium* (mineralised); l. *Celtis australis* (mineralised); m. *Prunus* subg. *Padus*. The scale bar is 1 mm; note the individual scale bars for *Celtis australis* and *Prunus* subg. *Padus*. See also Table S2.

3. Taphonomy

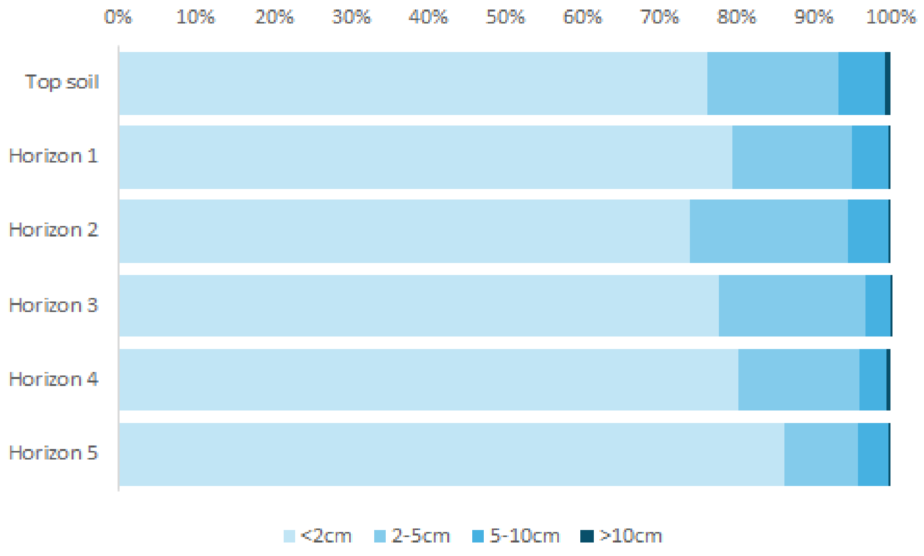

Figure S2: Bone and dental specimen lengths in the different archaeological Horizons in Yeghegis-1. The 3% (number of fragments (n)=304) of the bone fragments in the assemblage retained more than 50% of the total element length, and 7.7% (n=771) preserved more than 50% of the element's circumference. By contrast, 90.8% (n=9,124) and 89.4% (n=8,986) of the specimens preserved less than a quarter of the original length and complete element circumference. The 79.2% (n=7,958) of the analysed assemblage measured less than 20 mm, and 456 specimens (4.5%) measured larger than 50 mm.

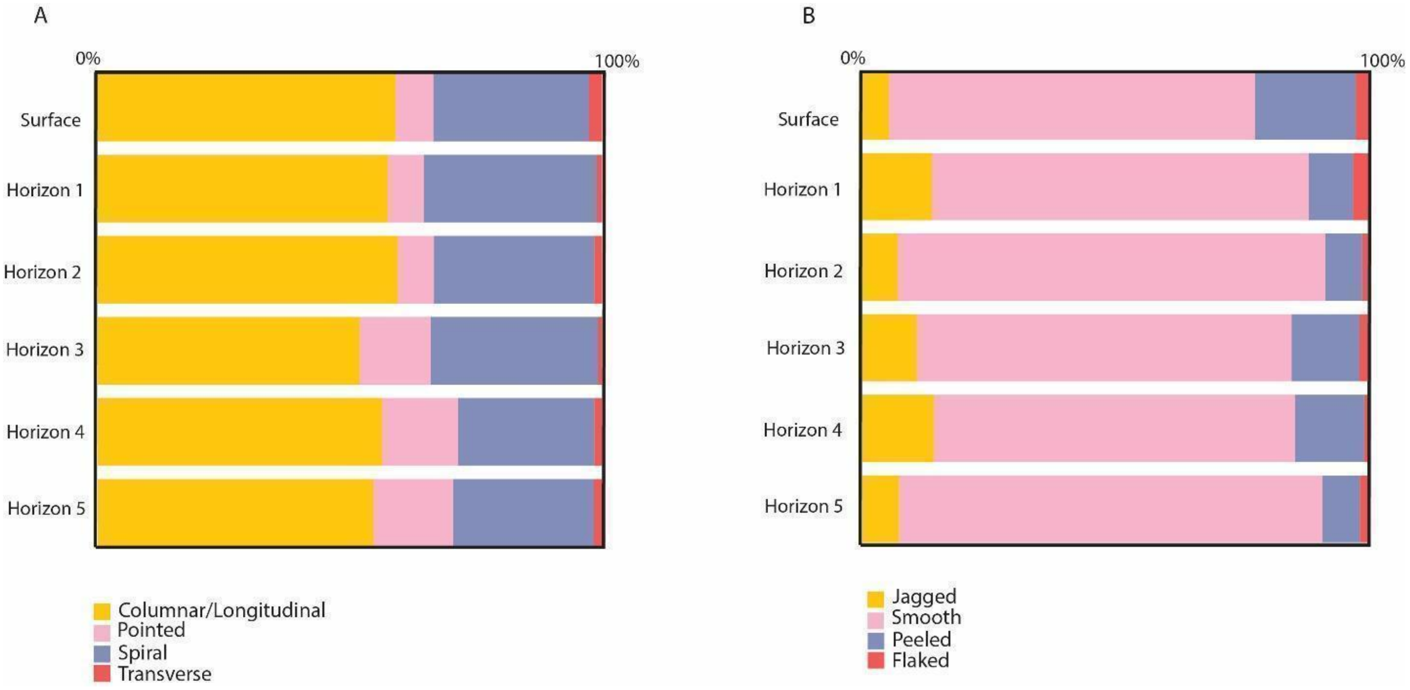

Figure S3: Bone fragmentation patterns (fracture outline and edge) observed in specimens from the different occupational Horizons in Yeghegis-1. A: Fragmentation outline, B: Fragmentation edge. The majority of the bone fragments exhibited columnar (56.7%) and spiral (30.8%) fragmentation outlines, mostly in a perpendicular (96.8%) angle and characterised by rather smooth (77.6%) fragmentation edges (Figure S3).

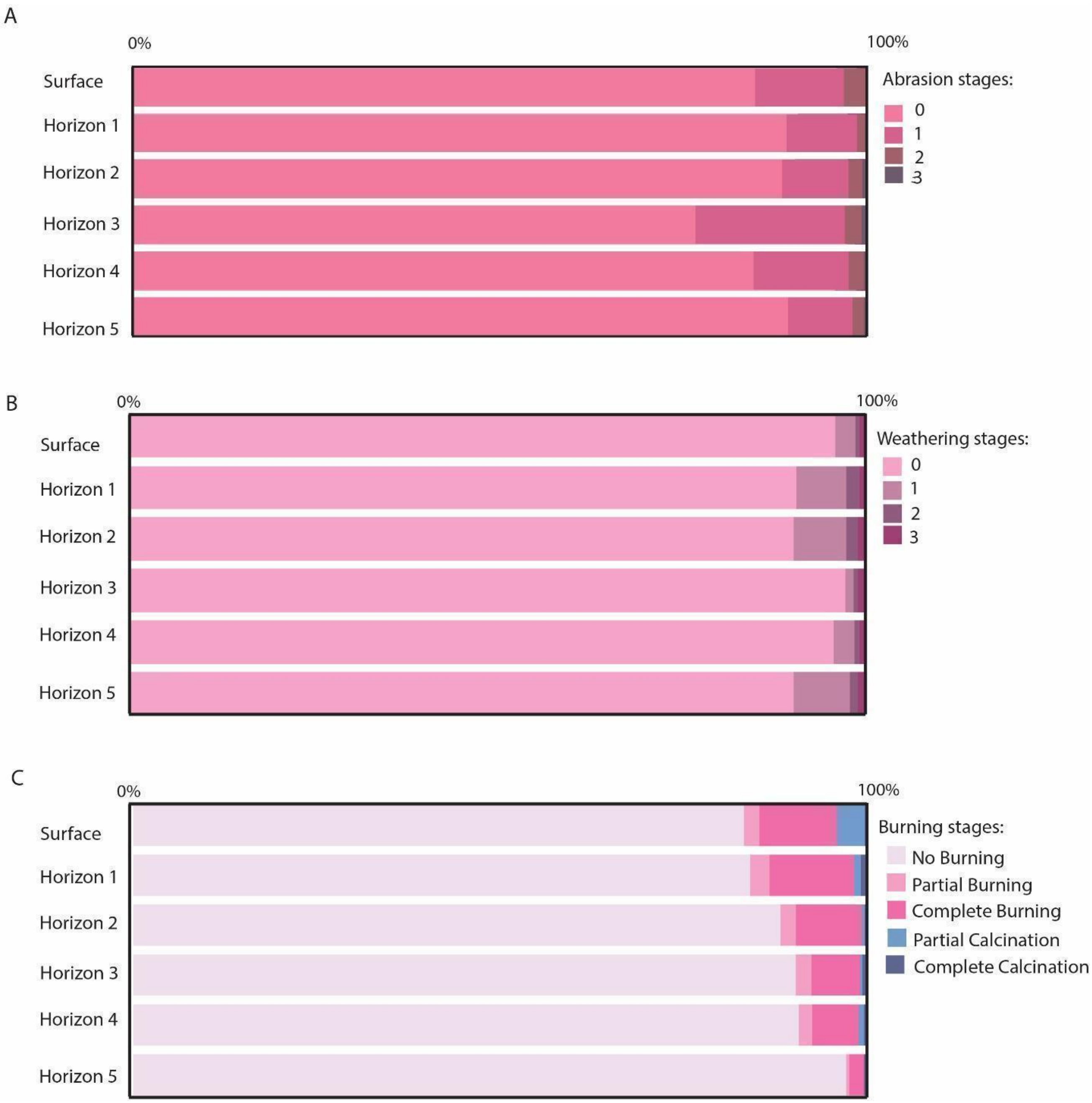

Figure S4: **Bone surface alterations (weathering, abrasion and burning) observed in the specimens from the different occupational horizons of Yeghegis-1.** A: Distribution of abraded specimens. B: Distribution of weathered specimens. C: Distribution of burnt specimens in the different archaeological Horizons in Yeghegis-1. Mid-to-heavy weathering was observed in 204 (1.9%) of the specimens examined. Mid to heavy abrasion was observed in only 231 (2.2%) of the specimens (Figure S4). Differential weathering was observed in low numbers (n=89, 0.86%). Burning (varying from partial burning to total calcination) was observed in 10.7% (n=1,109) of the bone fragments analysed, with some form of calcination observed in 105 specimens (1.01%). Evidence for butchery (cutmarks, chopmarks and scrapemarks) was observed in a total of 118 specimens (1.17% of total non-dental remains) (i.e. Figure 3). Evidence of carnivore modification (gnawing, canine pits and perforations as well as tooth scores) was recorded in a total of 248 (2.4%) bone specimens from the site (i.e. Figure 3). Bones with carnivore modifications were recovered in all archaeological Horizons, most notably in Horizon 2, where they were recorded in 2.8% of the specimens studied.

4. Morphological identifications

Table S3: The distribution of recovered skeletal remains per archaeological layer

Horizon	Unidentifiable bone	Identifiable bone	Teeth	Total
Horizon 0	494	57	5	**551**
Horizon 1	1367	139	13	**1506**
Horizon 2	2946	381	25	**3327**
Horizon 3	1537	142	14	**1679**
Horizon 4	1930	208	12	**2138**
Horizon 5	1064	131	8	**1195**
Total remains	**9338**	**1058**	77	**10396**

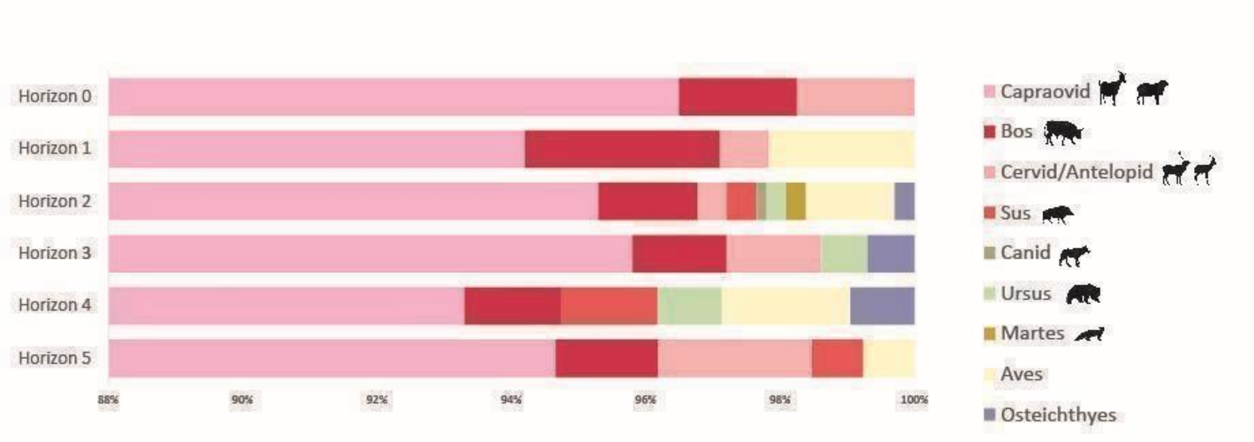

Figure S5: **Distribution of animal taxa identified in different Horizons.** In all Horizons Caprines dominate the record (n=989; 94.7 %). Remains of cattle were also recorded in all Horizons, albeit in very low frequency (n=25; 2.4 %). Other ungulates are represented by postcranial elements of representatives of Cervid/Antelopid (n=10; 0,9 %) family. Sus remains are also scarce (n=7; 0.7%) and were identified only in Horizons 2, 4 and 5. The morphological screening revealed one specimen of hare in Horizon 4. A rare occurrence of carnivores was recorded. In total, five (0.5%) bear remains, most probably *Ursus arctos*, were identified in Horizons 2, 3 and 4. A single *Canis* specimen was identified in Horizon 2, and a single *Vulpes* in Horizon 1, as well as two *Martes* remains were recovered from Horizon 2. Additionally, 17 bird specimens were identified in Horizons 1, 2, 4 and 5. The Zooarchaeological counts of identified remains per Horizon are represented in Table S4.

To investigate whether the taxonomic composition of Caprine remains varied across six stratigraphic horizons at the archaeological site, we conducted a chi-square test. Expected distributions were calculated under the null hypothesis that Caprine representation is uniform across horizons, proportional to the total number of bones recovered. The chi-square statistic (x^2= 9.01, p=0.109) indicated no significant difference in the relative proportion of Caprine remains among horizons when accounting for the total number of recovered bones. The analysis suggests that the relative abundance of Caprines is consistent across horizons, and any observed differences in raw counts are likely due to variations in recovery rates or sampling sizes rather than true changes in taxonomic representation.

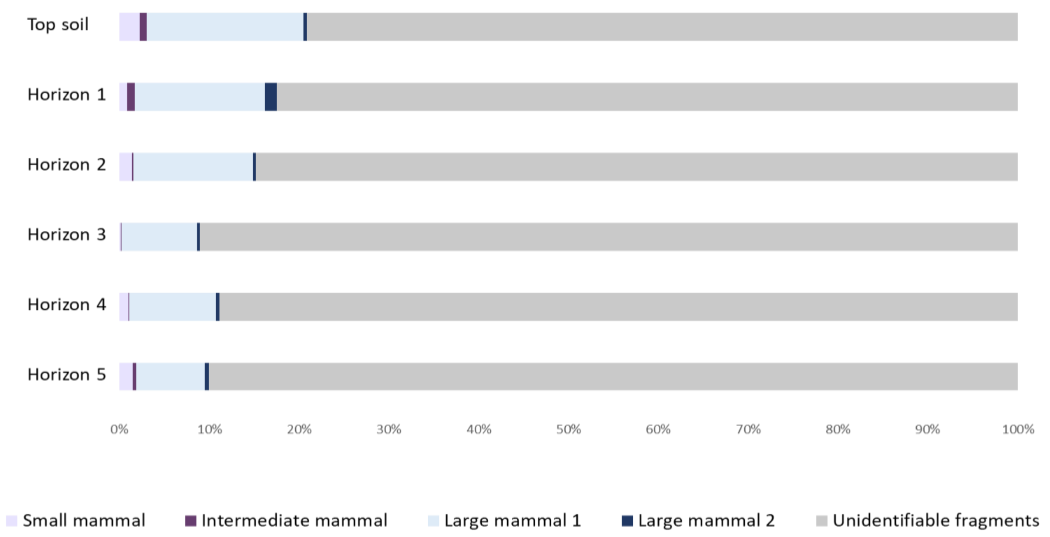

Figure S6: Distribution of unidentifiable specimens (n=1,247) assigned to size class (Table S4): small mammals (1 kg-10 kg); intermediate mammals (>10 kg-<50 kg, large mammals 1 (50-100 kg), large mammals 2 (>100 kg). A total of 1,075 bone fragments were grouped within the Large Mammal 1 class, which includes Artiodactyls of sheep/goat size. The Large Mammal 2 class group is considerably smaller, with only 44 specimens identified as being of the size of cattle or deer.

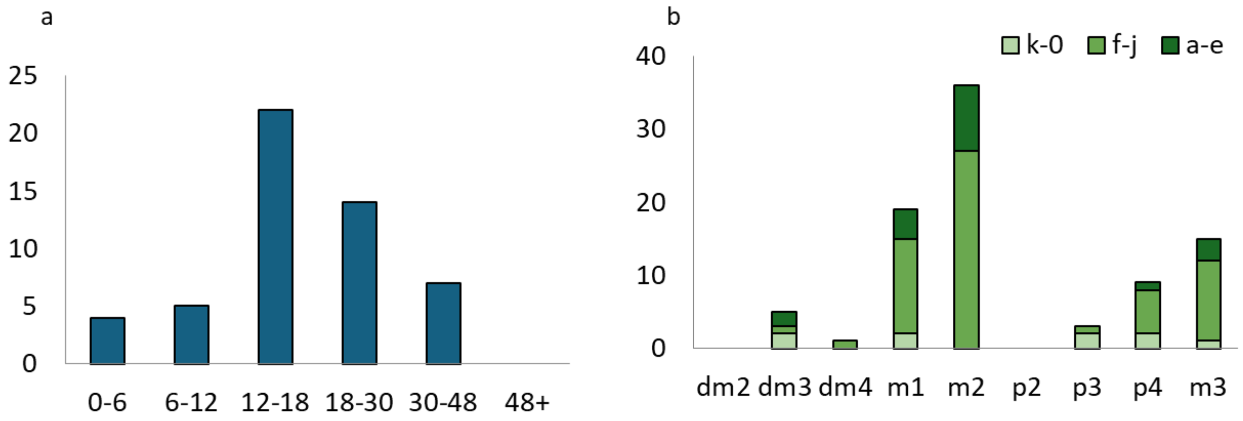

Figure S7: A: Age distribution from the unfused bones (in months) based on Zeder (2002)[11]. B: Dental wear stages: a-e (no to slight wear), f-j (moderate wear), and k-o (heavy wear) following the methodology established by Grant (1982)[12]. The teeth were classified by type, including deciduous molars, premolars, and molars (see Tables S10 and S11).

5. Zooarchaeology by Mass Spectrometry

Table S5: Distribution of samples selected for ZooMS screening across archaeological layers

**Horizon**	**Unidentifiable fragment <2 cm**	**Unidentifiable flat bone**	**Unidentifiable long bone**	**Morph ID-ed bone**	**Dental**	**Total**
Horizon 0	17	15	16	17	3	**68**
Horizon 1 (Subhorizon 1 & 2)	34	32	33	32	5	**136**
Horizon 2 (Subhorizon 1 & 2)	32	31	32	35	11	**142**
Horizon 3	18	16	17	17	7	**75**
Horizon 4 (Subhorizon 1 & 2)	36	34	33	33	5	**141**
Horizon 5	22	18	20	17	1	**78**

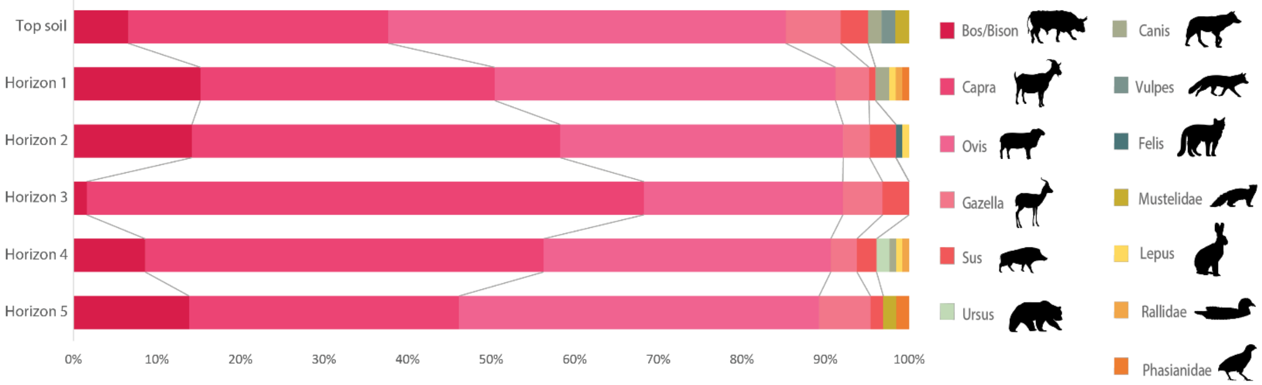

Figure S8: Distribution of taxa identified using ZooMS proteomic method. The faunal composition shows a predominance of Caprines, mainly represented by *Capra* (n=264) and *Ovis* (n=220). At the same time, other bovids are considerably less common *Bos* (n=65) and *Gazella* (n=24). The identified assemblage includes specimens of *Sus* (n=13) recovered from all Horizons. Carnivores are represented by *Ursus* (n=2), *Canis* (n=4), *Vulpes* (n=1) and *Felis* (n=1). Small mammalian composition is represented by *Lepus* (n=3) and Mustelidae (n=2). ZooMS allowed us to refine the taxonomy of the avian specimens to the family level for four specimens (Rallidae, n=2) and Phasianidae, n=2). The detailed ZooMS results can be found in Tables S6 and S7.

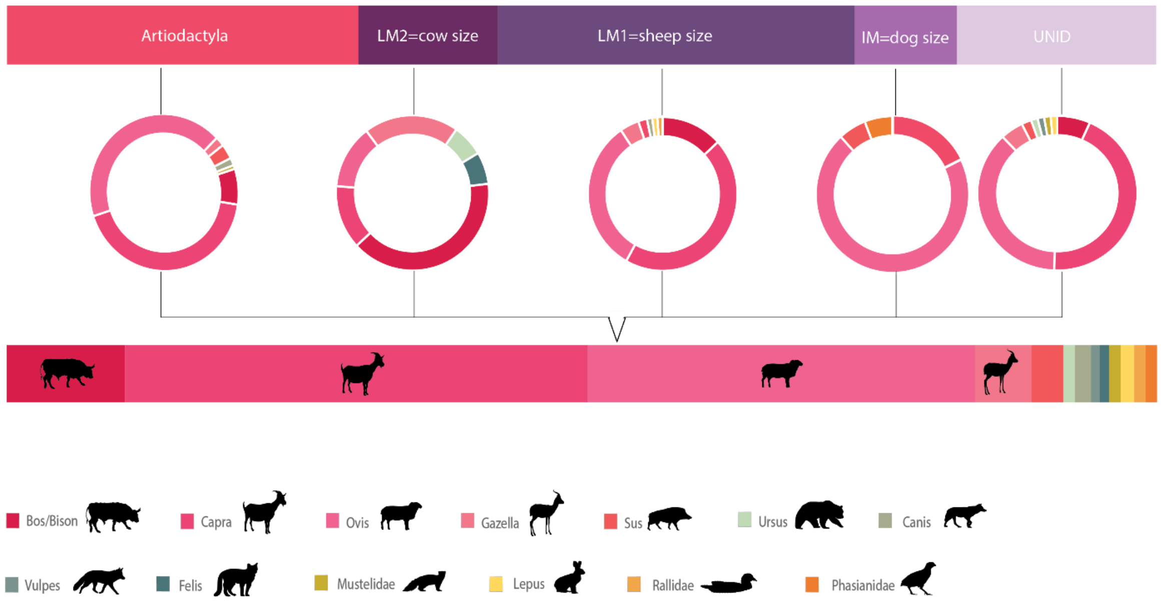

Figure S9: ZooMS identified taxa grouped by animal size classes. In many cases (20%), the body size estimates were not consistent with the ZooMS results.

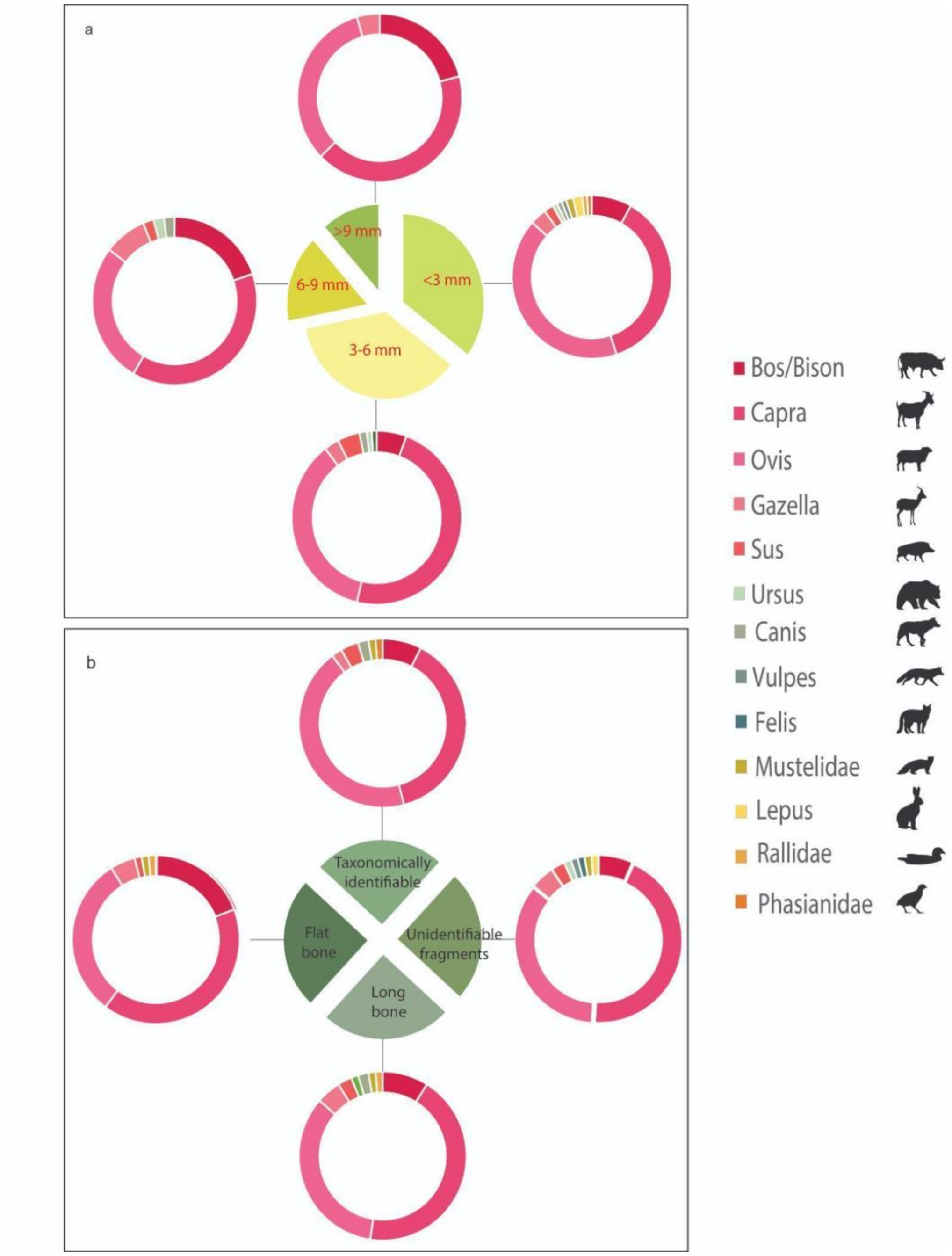

Figure S10: ZooMS identified taxa grouped by a) bone cortical thickness b) anatomical category: unidentifiable small bone fragments (<2 mm), unidentifiable long bone fragments, unidentifiable flat bone fragments, and anatomically identifiable specimens

6. Stable isotope analysis

Table S8: The distribution of animal remains sampled for isotope analyses across different archaeological layers

Horizon	Bulk enamel C/O	Sequential enamel C/O	Collagen C/N
Horizon 0	2	2	1
Horizon 1	3	3	2
Horizon 2	16	4	2
Horizon 3	6	3	3
Horizon 4	8	2	2
Horizon 5	6	-	3
Total	**41**	**14**	**13**

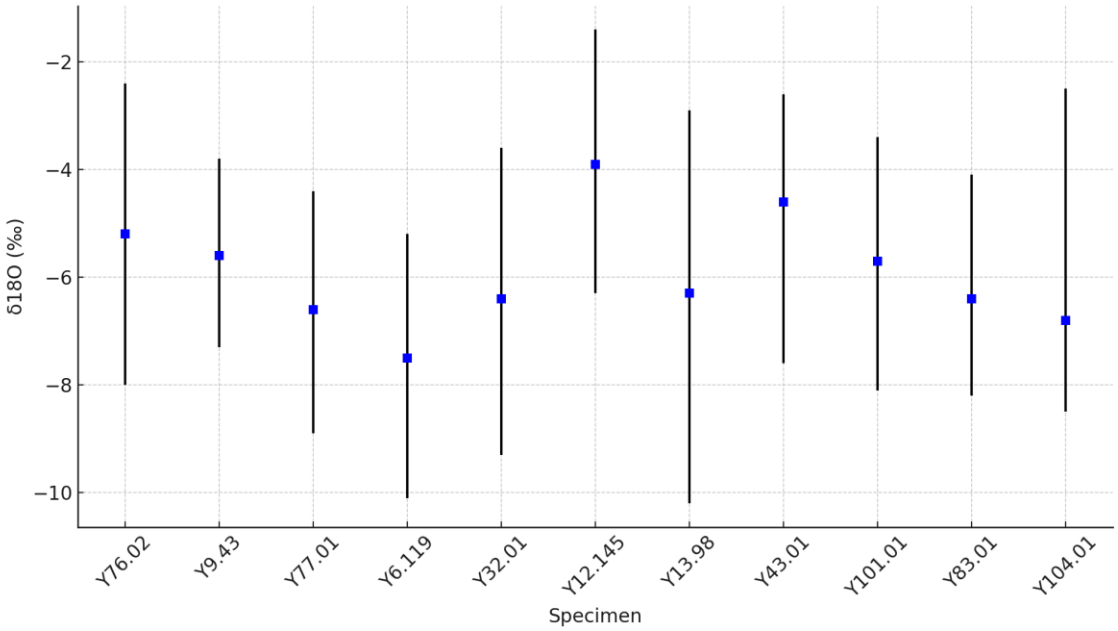

Figure S11: Comparison of δ¹⁸O mean, δ¹⁸O max, and δ¹⁸O min from incremental sheep and goat samples at Yeghegis-1 (n=11), including only specimens with sinusoidal variation, organized by Horizon. Isotope results are detailed in Table S11 and Figure 7. Sheep (Ovis, n=4) δ¹⁸O ranges: -10.1‰ to -3.8‰, with intra-tooth variation of 3.5‰–6.0‰. Goat (Capra, n=8) values span -10.2‰ to -1.4‰, with variation of 4.1‰–7.3‰. Sheep/goat (Ovis/Capra, n=2) show δ¹⁸O values from -10.1‰ to -2.7‰ and ~4.9‰ variation. Horizon-specific data show no significant difference in mean δ¹⁸O values (ANOVA p=0.79;

Kruskal-Wallis p=0.38). Specimens with sinusoidal patterns: Y101.01, Y104.01, Y12.145, Y13.98, Y.32.01, Y43.01, Y6.119, Y76.02, Y77.01. Attenuated/non-reliable patterns: Y12.149, Y31.01, Y4.70 (excluded). Y.9.43 shows sinusoidal variation with noise. The stable oxygen isotope composition of bioapatite closely reflects body water, as shown by Luz et al 1984)[13]. These values are influenced by ingested water, which in turn reflects meteoric $\delta^{18}O$, shaped by factors like altitude, humidity, and temperature[14,15]. Drinking water source matters: groundwater aligns with local $\delta^{18}O$ baselines, while river water varies seasonally due to meltwater. Evapotranspiration enriches $\delta^{18}O$ in leaf water above local levels[16]. The North Atlantic Oscillation (NAO) significantly impacts $\delta^{18}O$ in precipitation. Negative NAO phases weaken westerlies, allowing enriched Mediterranean-sourced precipitation to reach the Armenian Highlands; positive phases strengthen westerlies, bringing more depleted $\delta^{18}O$ from the Black Sea—most evident from December to May[17]. Altitude likely has limited $\delta^{18}O$ effect in the Caucasus Highlands[17]. Seasonal $\delta^{18}O$ reversal from snowmelt is unlikely, as observed summer values are too high to reflect typical winter $\delta^{18}O$ and vice versa.

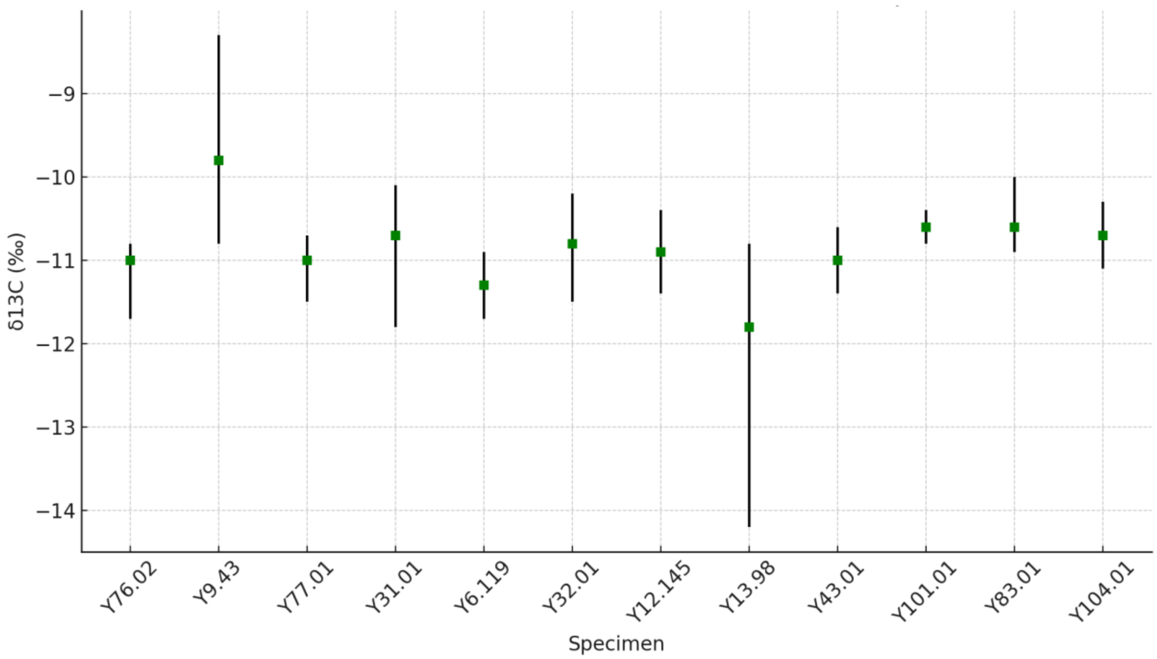

Figure S12. Comparison of $\delta^{13}C$ mean, $\delta^{13}C$ max, and $\delta^{13}C$ min from incremental sheep and goat samples at Yeghegis-1 (n=12), organized by Horizon. Sheep (n=4) $\delta^{13}C$ values range from -11.8‰ to -9.8‰, with intra-tooth variation of 0.8‰–2.5‰. The upper second molar (n=1) spans -11.8‰ to -10.1‰ (1.7‰ variation), the lower third molar (n=1) ranges from -10.8‰ to -8.3‰ (2.5‰), and the upper third molars (n=2) from -11.1‰ to -10.3‰ (0.4‰–0.8‰). Goat (n=8) values span -14.2‰ to -10.0‰, with 0.8‰–3.4‰ intra-tooth variation. Lower second molars (n=4) show 0.9‰–1.3‰ variation, lower third molar (n=1) varies by 3.4‰, upper second molars (n=2) by 0.8‰–0.9‰, and the upper third molar (n=1) by 0.8‰ (range: -11.5‰ to -10.7‰). Ovis/Capra (n=2) $\delta^{13}C$ values range from -12.0‰ to -10.2‰ (0.8‰ variation). The second upper molar (n=1) lacks a distinct minimum, while the upper third molar (n=1) ranges from -11.7‰ to -10.9‰ (0.8‰). Overall, $\delta^{13}C$ values show limited variation, indicating consistent C_3 plant consumption across the annual cycle. Notably, specimens Y9.43, Y76.02, Y77.01, Y4.70, Y31.01, Y6.119, Y32.01, Y12.149, and Y83.01 exhibit slight $\delta^{13}C$ increases during winter $\delta^{18}O$ minima (see Figure 7). Specimen Y13.98 shows the lowest $\delta^{13}C$ value (-14.2‰), suggesting a possible canopy effect and winter foraging in forested areas.

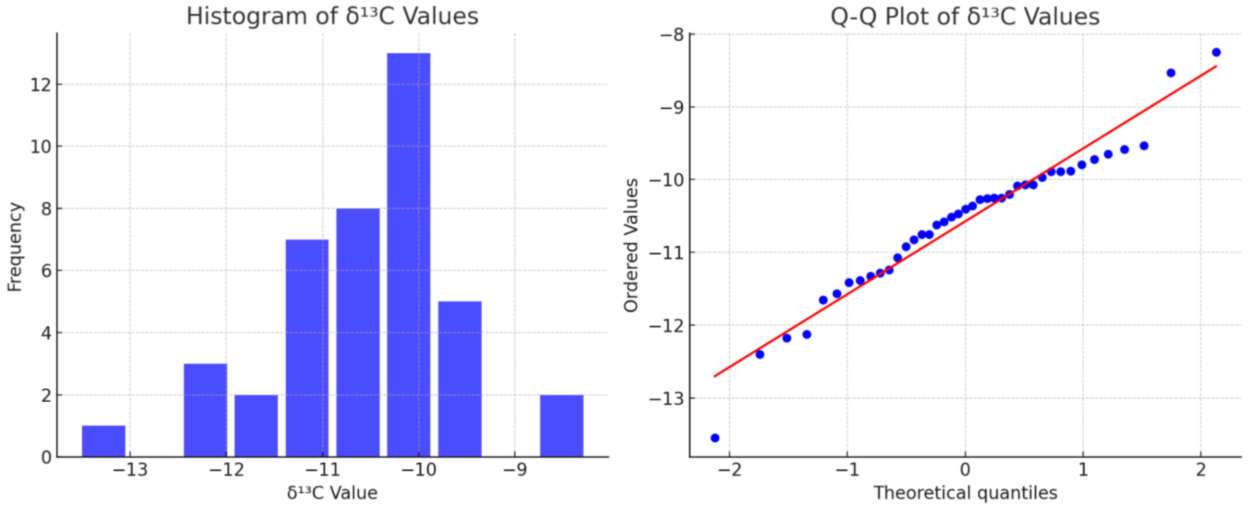

Figure S13. Histogram and Q-Q plot of δ¹³C values (n=41). The histogram shows the frequency distribution of δ¹³C values, while the Q-Q plot compares the observed data to a theoretical normal distribution. The alignment of points along the reference line in the Q-Q plot indicates no significant deviation from normality, supporting the results of the Shapiro-Wilk test (p = 0.198). To compare δ¹³C values across horizons (H0–H5), the data were first assessed for suitability for parametric testing. The Shapiro-Wilk test indicated no significant deviation from normality (p = 0.198), and Levene's test confirmed the homogeneity of variances (p = 0.140). Based on these results, a one-way ANOVA was performed, which showed no statistically significant differences in δ¹³C values between the horizons 0 - 5 (p = 0.332).

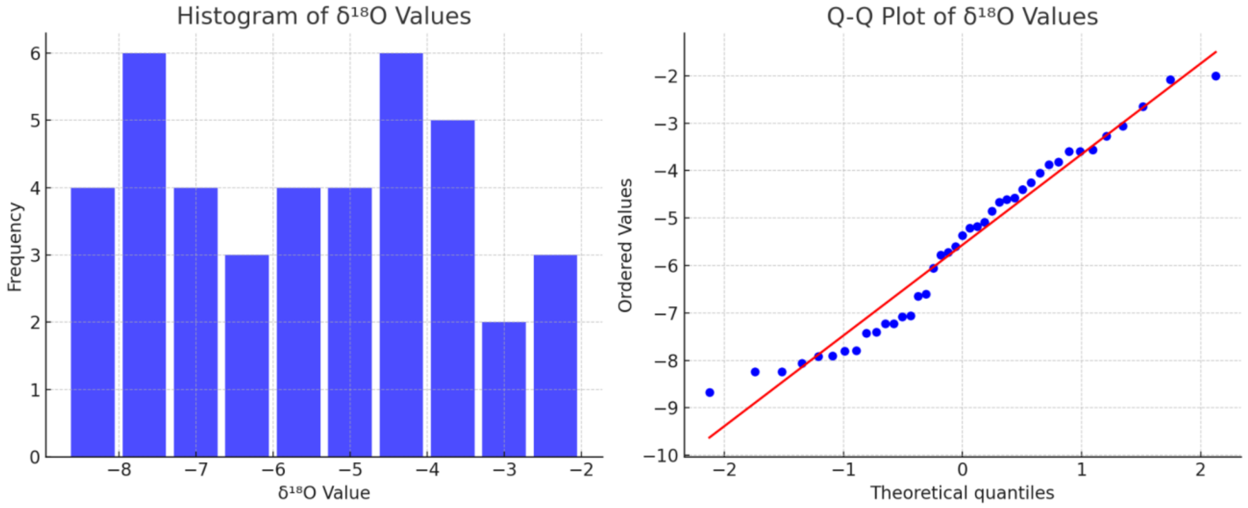

Figure S14: Histogram and Q-Q plot of δ¹⁸O values (n=41). The histogram (left) shows the distribution of δ¹⁸O values, while the Q-Q plot (right) indicates that the data aligns closely with a theoretical normal distribution, supporting the results of the Shapiro-Wilk test. To compare δ¹⁸O values across horizons (H0–H5), the data were first assessed for suitability for parametric testing. The Shapiro-Wilk test indicated no significant deviation from normality (p = 0.096), and Levene's test confirmed the homogeneity of variances (p = 0.278). Based on these results, a one-way ANOVA was performed. The one-way ANOVA returned a p-value close to the significance threshold (p = 0.060), indicating no statistically significant differences but suggesting a possible trend.

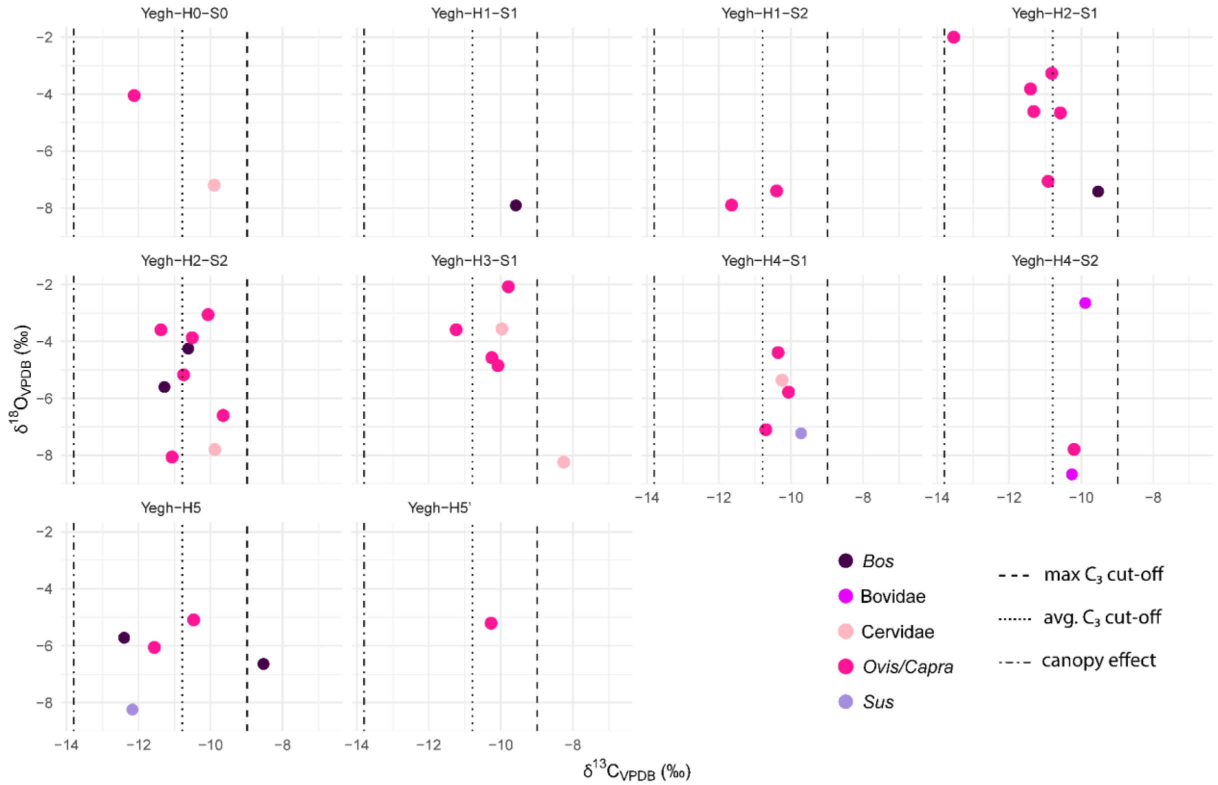

Figure S15. Bulk $\delta^{18}O$ and $\delta^{13}C$ isotope analysis of tooth enamel from *Bos* (n=6), Bovidae (n=2), Cervidae (n=5), *Ovis/Capra* (n=25), and Suidae (n=2) from Horizons 0–5 at Yeghegis-1. The maximum C_3 cut-off is based on local archaeological $\delta^{13}C$ values in C_3 plants from Yeghegis-1 (Table S1), while the average C_3 cut-off reflects global $\delta^{13}C$ values in C_3 plants[20]. A $\delta^{13}C$ value of -14.5‰ in ruminant enamel corresponds to a canopy-effect plant diet, based on modern (-29.7‰) and archaeological (-28.2‰) values[21]. Bos specimens show $\delta^{13}C$ values from -12.4‰ to -8.5‰ (average -10.3‰); Bovidae from -10.3‰ to -9.9‰; Cervidae from -10.3‰ to -8.3‰ (average -9.7‰); Ovis/Capra from -13.5‰ to -9.7‰ (average -10.8‰); and Suidae from -12.2‰ to -9.7‰. Bulk $\delta^{18}O$ values across all taxa range from -8.7‰ to -2.0‰. Bos $\delta^{18}O$ values range from -7.9‰ to -4.3‰ (average -6.3‰); Bovidae from -8.7‰ to -2.7‰; Cervidae from -8.2‰ to -3.6‰ (average -6.4‰); Ovis/Capra from -8.1‰ to -2.0‰ (average -5.1‰); and Suidae from -8.2‰ to -7.2‰. These data (see also Figures S14 and S15) suggest relatively stable environmental and ecological conditions throughout Horizons 0–5, with no clear long-term trends in climate or herding practices. Radiocarbon dating is discussed in Antonsyan et al. (2024)[18] and Frahm et al. (2024)[19].

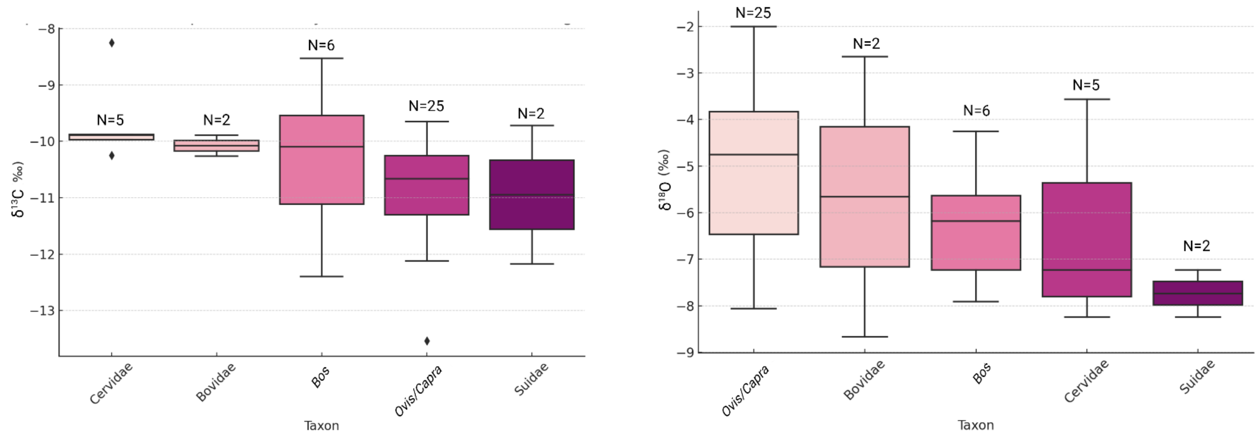

Figure S16. Boxplots of stable $\delta^{13}C$ (n=41) and stable $\delta^{18}O$ (n=41) isotope values from bulk enamel per taxon, sorted by mean from high to low.

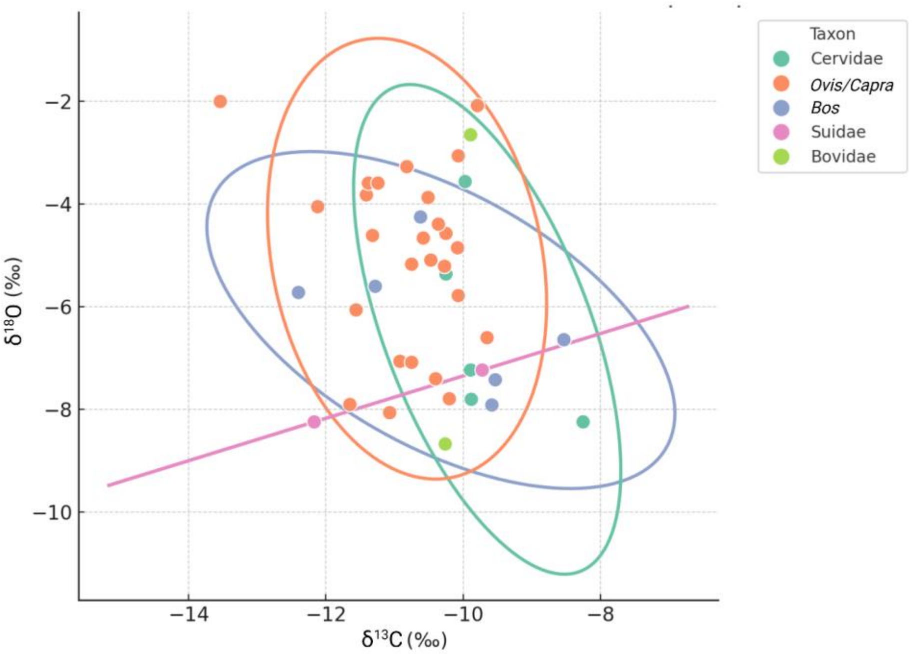

Figure S17: **Bivariate plot of bulk enamel stable $\delta^{13}C$ (n=41) and stable $\delta^{18}O$ (n=41) isotope values for *Bos,* Bovidae, Cervidae*, Ovis/Capra and Sus*.** Each data point represents an individual sample, color-coded by taxon, with 95% confidence ellipses overlaid to show the variation and overlap in isotopic signatures across the different taxa.

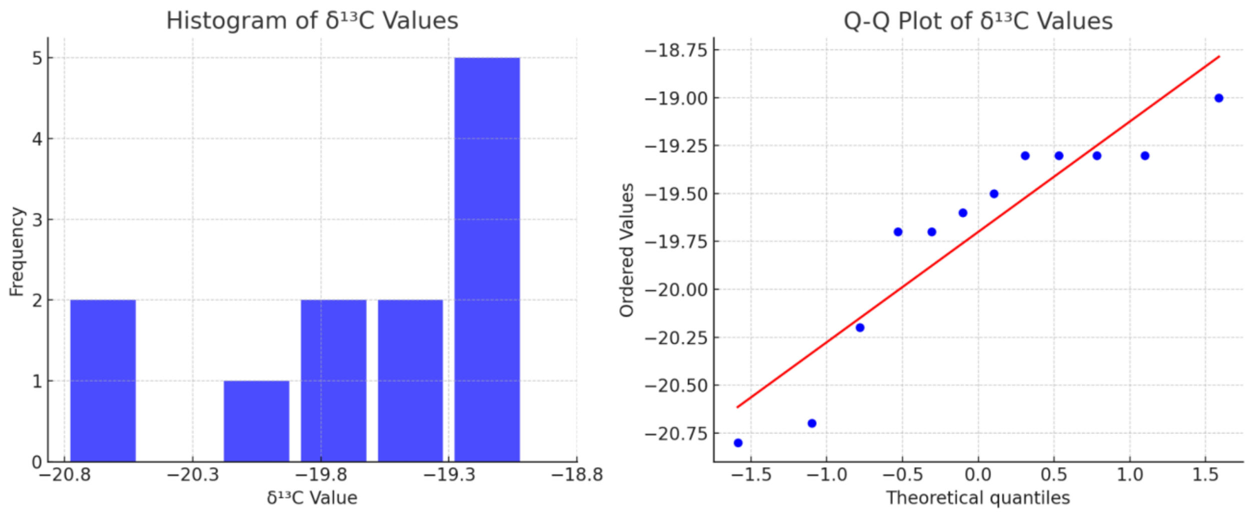

Figure S18. Histogram and Q-Q plot of δ¹³C values (n=13) used to assess normality. The histogram shows clustering and gaps, indicating a non-uniform distribution. The Q-Q plot reveals deviations from the normal line, especially at the distribution tails. The Shapiro-Wilk test ($p = 0.044$) rejects the null hypothesis of normality at the 5% significance level. Given the small sample size and non-normal distribution, the Kruskal-Wallis test was applied instead of ANOVA. The result ($p = 0.230$) indicates no significant difference in δ¹³C values across horizons.

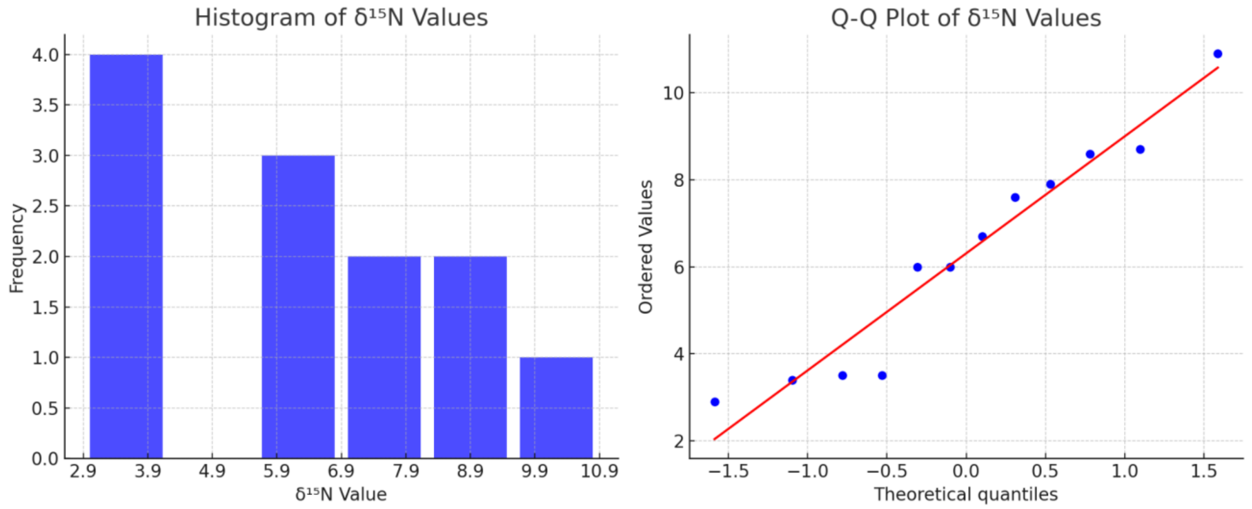

Figure S19. Histogram and Q-Q plot of δ¹⁵N values (n=13) used to assess normality. The histogram shows a skew toward higher values, while the Q-Q plot reveals some deviation from the normal line. However, the Shapiro-Wilk test ($p = 0.375$) does not reject the null hypothesis of normality at the 5% significance level. To test variance homogeneity across groups, Levene's test was applied and found no significant difference in variances ($p = 0.977$), indicating that the assumption of equal variances was met.

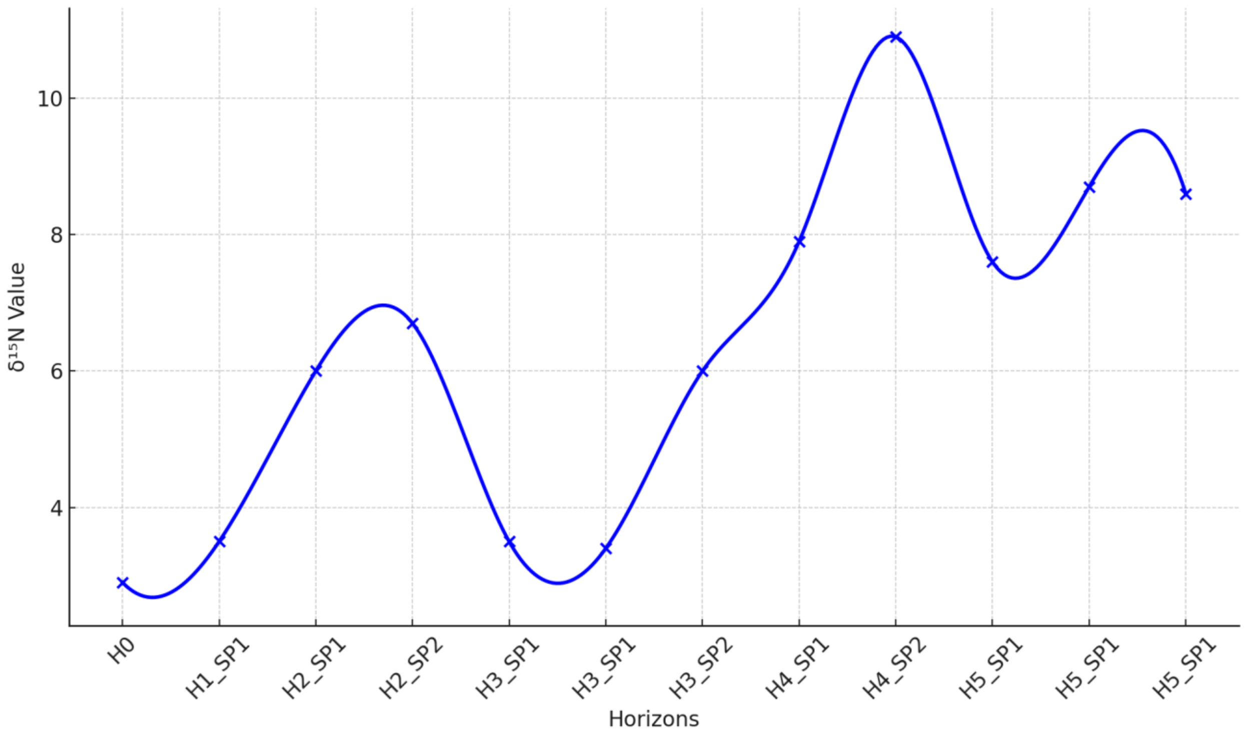

Figure S20. δ¹⁵N values (n=13) from caprines across Horizons 1–5 at Yeghegis-1 (see Table S9). The data show a progressive increase in δ¹⁵N values, particularly from Horizons 3 to 5, suggesting potential shifts in environmental conditions, diet, land use, or herding strategies over time. A one-way ANOVA was used to compare δ¹⁵N values across horizons (H1 SP1 to H5 SP1), with assumptions of normality, homogeneity of variances, and independence confirmed prior to analysis. The ANOVA yielded a statistically significant result ($p = 0.0067$), indicating differences in mean δ¹⁵N values across horizons.

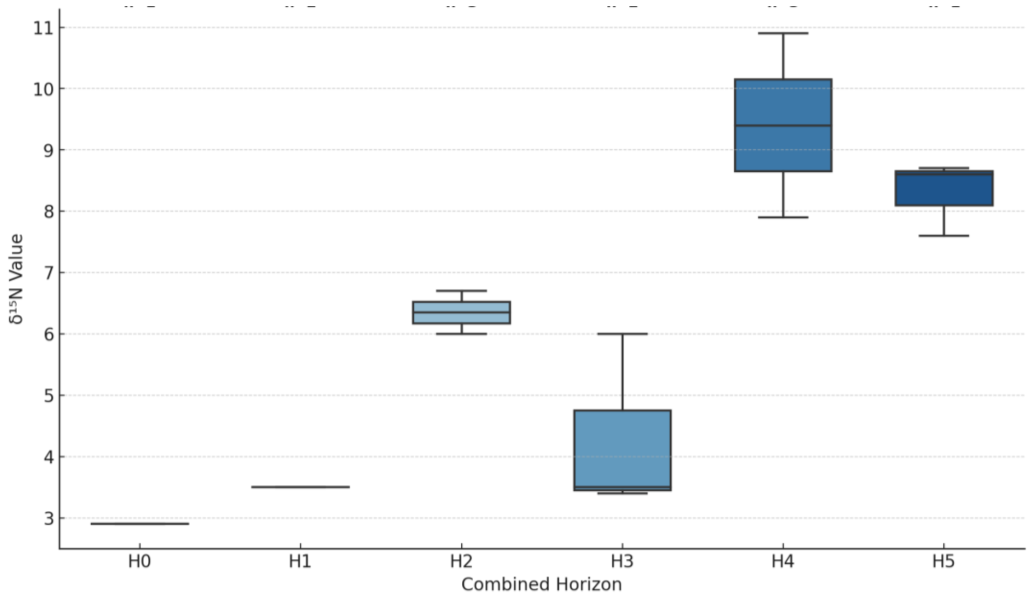

Figure S21: Boxplot of δ¹⁵N values by the horizon. δ¹⁵N values are shown for each horizon.
Horizons H3 and H5 (n = 3) and H4 (n = 2) display moderate to high variability, while H0 and H1 (n = 1) show no spread due to the limited sample size.

7. Paleoclimatic dataset

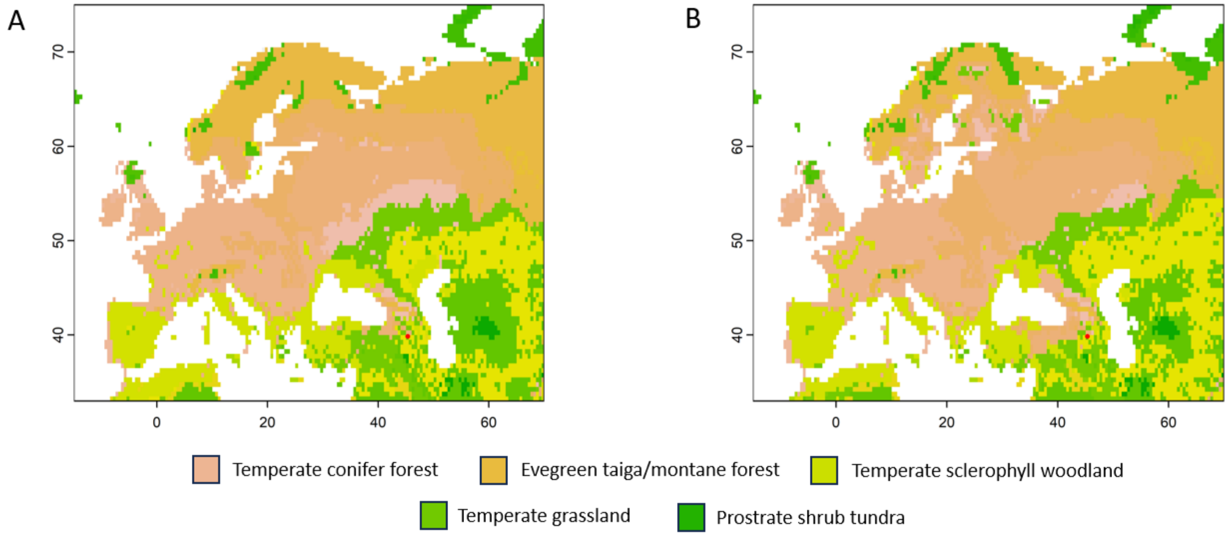

Figure S22: Biome distributions in Europe at 4050 BCE (A) and 3050 BCE (B). The red dot indicates the location of the Yeghegis-1 site. The dataset used covers the last 120,000 years at intervals of 1,000 years[22]. It utilises the Global Circulation Models HadCM3[23] and HadAM3H[24].

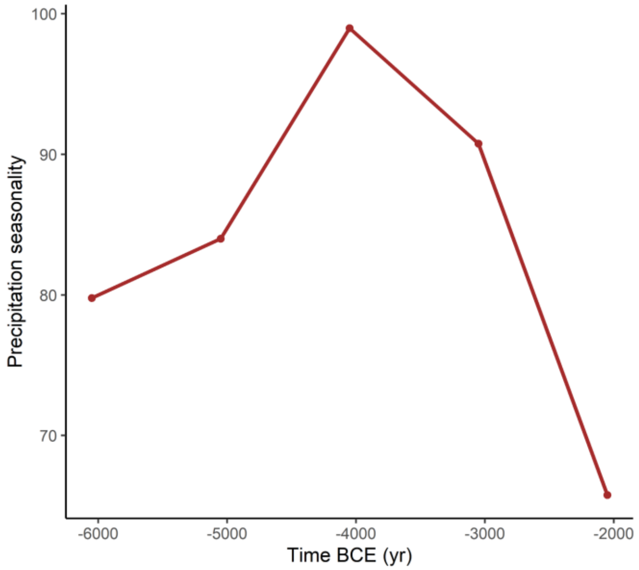

Figure S23: Precipitation seasonality in the site location according to the palaeoclimatic estimates. The values refer to the coefficient of variation of monthly precipitation.

Supplemental references

1. Spengler, R.N., Agriculture in the central Asian bronze age. Journal of World Prehistory, 28, 215-253 (2015)
2. Salzman, P. C., Multi-resource nomadism in Iranian Baluchistan. In W. Irons & N. Dyson-Hudson (Eds.), Perspectives on nomadism, 60–68. Leiden: E. J. Brill (1972)

3. Hovsepyan, R. and Willcox, G., The earliest finds of cultivated plants in Armenia: evidence from charred remains and crop processing residues in pisé from the Neolithic settlements of Aratashen and Aknashen. Vegetation history and archaeobotany, 17, 63-71 (2008)
4. Berthon, R., Decaix, A., Kovács, Z.E., Van Neer, W., Tengberg, M., Willcox, G. and Cucchi, T., A bioarchaeological investigation of three late Chalcolithic pits at Ovçular Tepesi (Nakhchivan, Azerbaijan). Environmental Archaeology, 18(3), 191-200 (2013)
5. Allué, E., Cáceres, I., Expósito, I., Canals, A., Rodríguez, A., Rosell, J., de Castro, J.M.B. and Carbonell, E., Celtis remains from the lower pleistocene of Gran Dolina, Atapuerca (Burgos, Spain). Journal of Archaeological Science, 53, 570-577 (2015)
6. Antonosyan, M., Roberts, P., Aspaturyan, N., Mkrtchyan, S., Lucas, M., Boxleitner, K., Jabbour, F., Hovhannisyan, A., Cieślik, A., Sahakyan, L. and Avagyan, A., Multiproxy evidence for environmental stability in the Lesser Caucasus during the Late Pleistocene. Quaternary Science Reviews, 330, 108559 (2024)
7. Xu, Z. and Deng, M., 2017. Identification and Control of Common Weeds: Volume 2. Dordrecht: Springer Netherlands.
8. Kakinuma, Kaoru; Okayasu, Tomoo; Sasaki, Takehiro; Jamsaran, Undarmaa; Okuro, Toshiya; Takeuchi, Kazuhiko . (2013). Rangeland management in highly variable environments: Resource variations across the landscape mediate the impact of grazing on vegetation in Mongolia. Grassland Science, 59(1), 44–51. doi:10.1111/grs.12008
9. Reinecke, J., Ashastina, K., Kienast, F., Troeva, E. and Wesche, K., 2021. Effects of large herbivore grazing on relics of the presumed mammoth steppe in the extreme climate of NE-Siberia. Scientific Reports, 11(1), p.12962.
10. Ying, T., Rihan, H., Hai, Y., Purevtseren, M. and Hoshino, B., 2018. Impact of settlement-type grazing on rangeland vegetation in the Inner Mongolia autonomous region: A field verification of case study through a grazing experiment. Heliyon, vol. 9, Issue 7, e17814
11. Zeder, M. A., Reconciling rates of long bone fusion and tooth eruption and wear in sheep (Ovis) and goat (Capra). Recent advances in ageing and sexing animal bones, 9, 87-118 (2006)
12. Grant, A., The use of tooth wear as a guide to the age of domestic animals. Ageing and sexing animal bones from archaeological sites, 91-108 (1982)
13. Luz, B., Kolodny, Y. & Horowitz, M. Fractionation of oxygen isotopes between mammalian bone-phosphate and environmental drinking water. Geochim. Cosmochim. Acta 48, 1689–1693 (1984).
14. Gat, J.R. Oxygen and hydrogen isotopes in the hydrologic cycle. Annu. Rev. Earth Planet. Sci. 24, 225–262 (1996).
15. Dansgaard, W. Stable isotopes in precipitation. Tellus 16, 436–468 (1964).
16. Pederzani, S. & Britton, K. Oxygen isotopes in bioarchaeology: principles and applications, challenges and opportunities. Earth-Sci. Rev. 188, 77–107 (2019)
17. Brittingham, A., Petrosyan, Z., Hepburn, J.C., Richards, M.P., Hren, M.T. & Hartman, G. Influence of the North Atlantic Oscillation on δD and δ18O in meteoric water in the Armenian Highland. J. Hydrol. 575, 513-522 (2019).
18. Antonosyan, M., Saribekyan, M., Mkrtchyan, S., Hovhannisyan, A., Frahm, E., Roberts, P., Bobokhyan, A., Azatyan, K., Yepiskoposyan, L. & Amano, N. Yeghegis-1 rockshelter site: new investigations into the late Chalcolithic of Armenia. Antiquity 98, 1–8 (2024).
19. Frahm, E., Saribekyan, M., Mkrtchyan, S., Furquim, L., Avagyan, A., Sahakyan, L., Azatyan, K., Roberts, P., Fernandes, R. & Yepiskoposyan, L. Increasing obsidian diversity during the Chalcolithic Period at Yeghegis-1 Rockshelter (Armenia) reveals shifts in land use and social networks. Sci. Rep. 14, 9528 (2024).
20. Kohn, M.J. Carbon isotope compositions of terrestrial C3 plants as indicators of (paleo) ecology and (paleo) climate. Proc. Natl Acad. Sci. USA 107, 19691-19695 (2010).
21. Balasse, M., Bălăşescu, A., Janzen, A., Ughetto-Monfrin, J., Mirea, P. and Andreescu, R., 2013. Early herding at Măgura-Boldul lui Moş Ivănuş (early sixth millennium BC, Romania): environments and seasonality from stable isotope analysis. European Journal of Archaeology, 16(2), pp.221-246.
22. Beyer, R.M., Krapp, M.,. & Manica, High-resolution terrestrial climate, bioclimate and vegetation for the last 120,000 years. Scientific data, 7(1), 236 (2020).
23. Singarayer, J. S. &Valdes, P. J., High-latitude climate sensitivity to ice-sheet forcing over the last 120 kyr. Quat. Sci. Rev. 29: 43–55 (2010).

24. Valdes, P. J., Armstrong, E., Badger, M. P. S., Bradshaw, C. D., Bragg, F., Crucifix, M., Davies-Barnard, T., Day, J., Farnsworth, A., Gordon, C., Hopcroft, P. O., Kennedy, A. T., Lord, N. S., Lunt, D. J., Marzocchi, A., Parry, L. M., Pope, V., Roberts, W. H. G., Stone, E. J., Tourte, G. J. L., Williams, J. H. T., The BRIDGE HadCM3 family of climate models: HadCM3@Bristol v1.0. Geosci. Model Dev. 10: 3715–3743 (2017).
